# Stemness marker ALDH1A1 promotes tumor angiogenesis via retinoic acid/HIF-1α/VEGF signalling in MCF-7 breast cancer cells

**DOI:** 10.1186/s13046-018-0975-0

**Published:** 2018-12-12

**Authors:** Valerio Ciccone, Erika Terzuoli, Sandra Donnini, Antonio Giachetti, Lucia Morbidelli, Marina Ziche

**Affiliations:** 1 Department of Life Sciences, University of Siena, Via A. Moro 2, 53100 Siena, Italy; 2 Department of Medicine, Surgery and Neuroscience, University of Siena, Via A. Moro 2, 53100 Siena, Italy

**Keywords:** Aldehyde dehydrogenase 1A1, Angiogenesis, Breast cancer cells, Stemness, Vascular endothelial growth factor

## Abstract

**Background:**

Aldehyde dehydrogenase 1A1 (ALDH1A1), a member of aldehyde dehydrogenase family, is a marker of stemness in breast cancer. During tumor progression cancer stem cells (CSCs) have been reported to secrete angiogenic factors to orchestrate the formation of pathological angiogenesis. This vasculature can represent the source of self-renewal of CSCs and the route for further tumor spreading. The aim of the present study has been to assess whether ALDH1A1 controls the output of angiogenic factors in breast cancer cells and regulates tumor angiogenesis in a panel of in vitro and in vivo models.

**Methods:**

Stemness status of breast cancer cells was evaluated by the ability to form turmorspheres in vitro. A transwell system was used to assess the angiogenic features of human umbilical vein endothelial cells (HUVEC) when co-cultured with breast cancer cells MCF-7 harboring different levels of ALDH1A1. Under these conditions, we survey endothelial proliferation, migration, tube formation and permeability. Moreover, in vivo*,* MCF-7 xenografts in immunodeficient mice allow to evaluate blood flow, expression of angiogenic factors and microvascular density (MVD).

**Results:**

In MCF-7 we observed that ALDH1A1 activity conferred stemness property and its expression correlated with an activation of angiogenic factors. In particular we observed a significant upregulation of hypoxia inducible factor-1α (HIF-1α) and proangiogenic factors, such as vascular endothelial growth factor (VEGF). High levels of ALDH1A1, through the retinoic acid pathway, were significantly associated with VEGF-mediated angiogenesis in vitro. Co-culture of HUVEC with ALDH1A1 expressing tumor cells promoted endothelial proliferation, migration, tube formation and permeability. Conversely, downregulation of ALDH1A1 in MCF-7 resulted in reduction of proangiogenic factor release/expression and impaired HUVEC angiogenic functions. In vivo, when subcutaneously implanted in immunodeficient mice, ALDH1A1 overexpressing breast tumor cells displayed a higher expression of VEGF and MVD.

**Conclusion:**

In breast tumors, ALDH1A1 expression primes a permissive microenvironment by promoting tumor angiogenesis via retinoic acid dependent mechanism. In conclusion, ALDH1A1 might be associated to progression and diffusion of breast cancer.

**Electronic supplementary material:**

The online version of this article (10.1186/s13046-018-0975-0) contains supplementary material, which is available to authorized users.

## Background

Angiogenesis plays an essential role in tumor progression by promoting the formation of tumor-associated neo-vessels [1]. Angiogenesis is instrumental for metastasis spreading, as it enables tumor cells entry into the circulatory system, and drives the formation of pre-metastatic vascular niche [2]. This functional unit contains endothelial cells (ECs), tumor cells and cancer stem cells (CSCs) which express and secrete a number of factors that activate stromal and ECs to grow and migrate, orchestrating the formation of the pathological vascular niches [2–4]. The CSC niche, characterized by hypoxia, has been reported to undergo a metabolic switch to a more invasive program [5], and to promote tumor neovascularization by producing high levels of proangiogenic factors such as vascular endothelial growth factor (VEGF), and interleukin 8 (IL-8). In this context, inhibition of EC responsiveness to angiogenic factors, and suppression of proangiogenic phenotype in tumor cells may attenuate aggressiveness of CSCs. Therefore, characterization of molecular signatures governing the cellular dynamics within these pathological niches may curb tumor burden. Aldehyde dehydrogenases (ALDHs), a family of NADP-dependent enzymes, catalyze the oxidation of a broad spectrum of toxic aldehydes. High levels of aldehyde dehydrogenase 1A1 (ALDH1A1) expression and activity have been proposed as a reliable CSC marker, since they are associated with cancer stem-like features, as cell self-protection, differentiation, expansion, and therapy resistance [6]. The potential role of ALDH1A1 in mediating the angiogenic phenotype in breast CSCs and tumor neovascularization is less known. ALDH1A1 isozyme oxidizes retinaldehyde to retinoic acid (RA). RA regulates the expression of a variety of genes through RAR and RXR nuclear receptors, that control the transcription of target genes possessing the RA response elements (RAREs) [7]. However, in CSCs from human melanoma, ALDH1A1 modulates either RA-driven target genes with RAREs and genes associated with aggressiveness/stem cell functions, making this isozyme a putative therapeutic target in melanoma [8]. In some breast cancer cell lines, the expression of RA-inducible genes affects tumor growth and metastasis [9]. In this work we determined whether ALDH1A1 in breast cancer cells was involved in the output of angiogenic factors and whether it might influence tumor angiogenesis in a number of in vitro and in vivo experimental settings. We identify a novel role of ALDH1A1 in some breast tumor cells lines, which, through RAR-dependent VEGF expression regulates tumor angiogenesis.

## Methods

### Chemicals and reagents

The ALDH1A1 selective inhibitor CM037 was from ChemDiv Inc. (San Diego, CA, USA). CM037 was dissolved in DMSO (10 mM) and subsequent dilutions were done in medium. Acetaldehyde, NADH, and cobalt chloride (CoCl_2_) were from Sigma (St. Louis, MO, USA).

CelLytic™ MT Cell Lysis Reagent, Fluoromount Aqueous Mounting Medium, 3 kDa FITC-Dextran were from Life Technologies (Carlsbad, CA, USA). Lentiviral particles were from OriGene (Rockville, MD, USA). Bevacizumab was kindly provided from University Hospital of Siena, Le Scotte. β-estradiol, streptavidin-conjugated HRP,3,3-diaminobenzidine tetrahydrocloride (DAB) and Eukitt® Quick-hardening mounting medium were from MerckKGaA (Darmstadt, Germany). β-estradiol was dissolved in cotton seed oil. Retinoic acid, pan-RAR antagonist (AGN193109) and RXR antagonist (UVI3003) were from Tocris Bioscience (Bristol, United Kingdom). Matrix Matrigel (growth factors and phenol red-free) was from Becton Dickinson (Waltham, MA, USA). Tissue-Tek O.C.T. was from Sakura (San Marcos, CA, USA).

Anti-ALDH1A1, anti-Ki67, anti-KLF4, anti-SOX2 and anti-VE-Cadherin antibodies were from Cell Signalling Technology (Danvers, MA, USA). Anti-VEGF, anti-β-Actin and anti-NG2 antibodies were from Merck KGaA (Darmstadt, Germany). Anti-HIF-1α and anti-CD31 antibodies were from BD Biosciences (Franklin Lakes, NJ, USA). Anti-CD133 was from Boster Biological Technology (Pleasanton, CA, USA). Secondary antibodies goat anti-rabbit IgG and anti-mouse were from Merck KGaA (Darmstadt, Germany). Goat anti-rat IgG Alexa Fluor 568 and goat anti-rabbit Alexa Fluor 488 antibodies were from Thermo Fisher Scientific (Waltham, MA, USA).

### Cell cultures

The human breast cancer adenocarcinoma MCF-7, MDA-MB-231 and SKBR-3 cells were obtained from the American Type Culture Collection. These cells represent different breast cancer histotypes. MCF-7 cells express estrogen receptors (ER), progestin receptor (PR) but lack of HER-2 receptor (HER2-). MDA-MB-231 cells represent triple negative model (ER-, PR-, HER2-). Finally, SKBR3 cells have HER2 amplification (HER2+). MCF-7 and MDA-MB-231 were maintained in DMEM High glucose (Euroclone, Milan, Italy) and SKBR-3 in RPMI 1640 (Euroclone, Milan, Italy) supplemented with 10% fetal bovine serum (FBS) (Hyclone, Celbio, Milan, Italy) and 2 mM glutamine, 100 units/ml penicillin and 0.1 mg/ml streptomycin (Merck KGaA, Darmstadt, Germany).

Human umbilical vein endothelial cells (HUVECs) were purchased from Promocell (Heidelberg, Germany) and were grown in endothelial growth medium (EGM-2), containing VEGF, R^3^-IGF-1, hEGF, hFGF, hydrocortisone, ascorbic acid, heparin and GA-1000 (Lonza, Basel, Switzerland), 10% FBS and 2 mM glutamine, 100 units/ml penicillin and 0.1 mg/ml streptomycin (Merck KGaA, Darmstadt, Germany). Cells were cultured at 37 °C in 5% CO_2_.

Breast cancer cells were split 1:4 twice a week, and used until passage 10. HUVEC cells between passage 3 to 5 were used in the experiments. Control of mycoplasma was performed from frozen vials. To achieve a stable knockdown, breast cancer cells were seeded on 6-multiplates and transduced at 70% confluence with lentiviral particles (Sigma) carrying a scrambled or two ALDH1A1 shRNA sequences (TRC N 0000276459 and TRC N 0000276397) and expressing the puromycin-resistant gene. Thirty-six hour post-infection, puromycin (2 μg/ml) was added to cells, and selection was allowed for 3 days. Cells were used in the experiment or split for propagation. Selected cells were maintained in complete DMEM medium with puromycin (1 μg/ml).

The sequence of plasmid inserted in cells clone 1 (ShA) is: 5′- CCGGCACCGATTTGAAGATTCAATACTCGAGTATTGAATCTTCAAATCGGTGTTTTTG.

The sequence of plasmid inserted in cells clone 2 (ShB) is: 5’-CCGGCTCTAGCTTTGTCATAGTTATCTCGAGATAACTATGACAAAGCTAGAGTTTTTG.

To generate a stable ALDH1A1 overexpression (ALDH1A1^+^), breast cancer cells were seeded on 6-multiplates and transfected with lentiviral particles containing nucleotide sequences encoding for ALDH1A1 (Origene RC200723 LentiORF particles, ALDH1A1 (Myc-DDK tagged) - Human). ALDH1A1^+^ cells were generated by G418 (400 μg/ml) selection for 10 days.

### Transfection of siRNAs

siRNAs targeting ALDH1A1 and HIF-1α used for transient knock-down experiments were purchased from Qiagen (Hilden, Germany). Cells were transfected with 20 nM targeting siRNA (two sequences) or scrambled control siRNA using Lipofectamine® RNAiMAX (Invitrogen) according to manufacturer’s instructions. Cells were assayed 48–72 h after transfection. Knockdown efficiency was assessed by immunoblotting or quantitative RT-PCR analysis.

### RNA isolation and quantitative RT-PCR

RNA isolation and quantitative RT-PCR (qRT-PCR) were performed on cell cultures and tissue samples. RNA extraction from tumor samples started with distruption and homogenization using the TissueLyser II (#85300 Qiagen). Total RNA was prepared using RNeasy Plus Kit (#74134 Qiagen) following manufacturer’s instructions. One μg RNA was reverse transcribed using QuantiTect Reverse Transcription Kit (#205313 Qiagen) and quantitative RT-PCR was performed using QuantiNovaSYBR Green PCR Kit (#208056 Qiagen) in a Rotor-Gene qPCR machine (Qiagen). Fold change expression was determined by the comparative Ct method (ΔΔCt) normalized to 60S Ribosomal protein L19 expression (Additional file 1: Table S1 for qPCR primer list). qRT-PCR data are represented as Ct value (cycle threshold) or fold increase relative to scrambled cells (Scr), which were assigned to 1.

### Western blot

Western blot was performed on cell culture lysates and tissue samples. Subconfluent breast cancer cells were seeded in 60 mm Petri dishes. After adherence, were indicated, cells were treated with retinoic acid (1 μM, 48 h), AGN193109 (1 μM, 48 h), UVI3003 (1 μM, 48 h), CM037 (1 μM and 10 μM for 18 h), CoCl_2_ (100 μM, 72 h), exposed to 1% or 10% FBS for 48 h. Proteins were isolated and western blots were performed as previously described [10]. Briefly, cells were washed 2x with cold Dulbecco’s Phosphate Buffered Saline (Sigma Aldrich) and lysed on ice with CelLytic™ MT Cell Lysis Reagent supplemented with 2 mM Na_3_VO_4_ and 1x Protease inhibitor cocktail for mammalian cells (Sigma Aldrich). Protein extraction from tumor samples started with disruption and homogenization using the TissueLyser II (#85300 Qiagen). Cell lysates (derived from cell cultures or tissues) were centrifuged at 16000×g for 20 min at 4 °C and the supernatants were then collected. Protein concentration was determined using the BCA protein assay kit (#23227 Thermo Fisher Scientific). Electrophoresis (50 μg of protein/sample) was carried out in 4–12% Bis-Tris Gels (Life Technologies, Carlsbad, CA, USA). Proteins were then blotted onto nitrocellulose membranes, incubated overnight with primary antibodies and then detected by enhanced chemiluminescence system (Biorad, Hercules, CA, USA). Results were normalized to those obtained by using an antibody against β-Actin.

All experiments were performed at least three times. Immunoblots were analyzed by densitometry using NIH Image J 1.48v software, and the results, expressed as arbitrary density units (A.D.U.) ± SD, were normalized to β-Actin.

### ALDH1A1 enzymatic activity

ALDH1A1 enzyme activity was determined by measuring the conversion of acetaldehyde to acetic acid, as reported [11]. Briefly, cells were cultured as above, then scraped into 600 μl lysis buffer (100 mM Tris-HCl pH 8.0, 10 mM DTT, 20% glycerol, 1% Triton X-100), and centrifuged at 16000×g for 20 min at 4 °C. The supernatant was used to detect ALDH activity at 25 °C by monitoring NADH formation from NAD^+^, at 340 nm in a spectrophotometer (Infinite F200 Pro, Tecan Life Sciences, Switzerland). The assay mixture (0.8 ml) contained 100 mM sodium pyrophosphate pH 9.0, 10 mM NAD^+^ and 600 μg of sample protein. The reaction was started by adding acetaldehyde (10 mM) to the cuvette. Enzyme-specific activity was expressed as nmol NADH/minute/mg protein.

### In vitro tumorsphere formation

This assay tests the ability of single cells to form tumorspheres, the in vitro surrogate of stem-like cell. Breast tumor cells (2 × 10^5^ cells/well in 1.5 ml of medium) were distributed into ultralow attachment 6-well plates. Tumorspheres were grown in DME-F12 medium (Gibco), supplemented with penicillin/streptomycin, L-glutamine and B27 supplement (1x, #17504–044, Life technologies), fibroblast growth factor-2 (FGF-2) (20 ng/ml, #13256029, Gibco) and hEGF (20 ng/ml, #10605-HNAE, Gibco), and allowed to grow for 7 to 10 days, or until the majority of spheres reached a diameter of 60 μm. Tumorspheres were counted and then harvested for protein extraction or splitted for second generation of tumorspheres and next lysed for protein extraction [12].

### MTT test

Tumor cell survival was quantified by MTT assay [13]. 3 × 10^3^ MCF-7 cells (Scr, ALDH1A1KD, ALDH1A1^+^) were seeded in 96-multiwell plates in medium with 10% serum and after adherence were exposed to different concentration of FBS (0.1 and 10%) or VEGF (2 and 20 ng/ml with 0.1% FBS medium) for 72 h. Medium was removed and cells were incubated for 4 h with fresh medium in the presence of 1.2 mM 3-(4,5-dimethylthiazol-2-yl)-2,5-diphenyltetrazolium bromide (MTT). After solubilization in DMSO, absorbance was measured with a microplate absorbance reader (Infinite 200 Pro, Tecan Life Sciences, Switzerland) at 540 nm. Data are reported as 540 nm relative absorbance/well.

### ELISA for VEGF levels

3 × 10^4^ MCF-7 cells (Scr, ALDH1A1KD and ALDH1A1^+^) were plated into 24-well plates. After adherence medium was changed with 1% FBS medium and cells were incubated for 48 h. In alternative, naïve MCF-7 were plated into 24-well plates. After adherence, cells were treated with CM037 (10 μM) for 18 h. Conditioned media were collected and VEGF levels were measured using an ELISA kit (R&D Systems, Minneapolis, MN, USA) following the manufacturer’s instructions. As a reference, cells were fixed, stained and counted.

### Human cytokine ELISA plate Array

We used the Human Cytokine ELISA Plate Array (#EA-4001, Signosis Inc., Santa Clara, Ca, USA), for quantitative comparison of 32 cytokines among different samples. Cells were exposed to medium with 1% FBS in presence/absence of CM037 (1 μM) for 48 h (with CM037 treatment every 24 h). The cell culture supernatants from each sample were incubated in the wells of the cytokine ELISA plate, and the captured cytokine proteins were subsequently detected with a cocktail of biotinylated detection antibodies. The test sample was allowed to react with a pair of antibodies, resulting in the cytokines being sandwiched between the solid phase and enzyme-linked antibodies. After incubation, the wells were washed to remove unbound-labelled antibodies. The plate was further detected with HRP luminescent substrate. The level of expression for each specific cytokine is directly proportional to the luminescence intensity. Data are reported as % of fold change vs. untreated cells. The experiment was performed 2 times in duplicate.

### In vivo tumor xenograft

Investigation has been conducted in accordance with the ethical standards and according to the Declaration of Helsinki and the Italian law (Legislative Decree no.26, 4 March 2014), which acknowledges the European Directive 2010/63/UE, being approved by the authors’ institutional review board and the Italian Ministry of Health. To assess the involvement of ALDH1A1 in tumor growth and angiogenesis, immunodeficient mice (5 week-old female athymic mice, Envigo Huntingdon, UK) were s.c. inoculated in the right flank with 10^7^ MCF-7 cells/100 μl (50 μl of cells and 50 μl of Matrigel). Eighteen different mice were randomly assigned to 3 different groups of 6 mice. The first group mice were injected with MCF-7 Scr. MCF-7 ALDH1A1KD and MCF-7 ALDH1A1^+^ were injected respectively in the second and third group. β-estradiol was administered i.m. (3 mg/kg), every 7 days. Mice were daily observed and caliper measurements of tumors were done twice a week. During experiments mouse weight did not change. Tumor dimensions were determined using the following formula: (shortest diameter × longest diameter × thickness of the tumor in mm). Data are reported as tumor volume in mm^3^. After 23 days mice were sacrificed. For each tumor, volume (mm^3^) and weight (mg) were determined. Each tumor was collected and split in two parts. One part was immediately frozen in liquid nitrogen for Western blot and RT-PCR analysis. Each tumor sample obtained was halved, one for RT-PCR analysis and one for Western blot. Each sample was then analyzed at least two times. The other part was embedded in Tissue-Tek O.C.T., cooled in isopentane and frozen in liquid nitrogen for histology. Seven-μm-thick cryostat sections from tissue samples were used for immunohistochemical staining with anti-Ki67 antibody and immunofluorescence with anti-CD31 and anti-NG2 antibodies [14].

### Three-dimensional ultrasound high resolution tumor imaging

Ultrasound imaging was done using Vevo 2100 Imaging System (Visual Sonics Inc., Toronto, ON, Canada) to assess relative perfusion in a tumor area or a tumor volume.

Mice were anesthetized with 2% isofluorane in oxygen and restrained on a heated stage (THM-100, Indus Instruments, Houston, TX) during imaging. Ultrasound coupling gel (Aquasonic 100, Parker Laboratories, Inc., Fairfield, NJ) was applied to the skin between the transducers and the mice, and images of the tumors were acquired through the ventral body wall in longitudinal orientation. Power Doppler Mode has been used to visualize and measure flow in 3D. This mode allows to detect vascularity in and around subcutaneous tumors and produces a measure of relative quantification as percent vascularity, an index of relative vascular density. Positive areas appear in red. Power 3D-Mode, added to Power Doppler Mode, allows to reconstruct a volume that integrates the Power Doppler Mode color data with the surrounding B-Mode 3D volume.

### Immunohistochemistry staining on O.C.T. sections

Seven-μm-thick cryostat sections from tissue samples were used for immunohistochemical staining with anti-Ki67 antibody. Cryostat sections were firstly fixed in 4% paraformaldehyde (PFA) for 20 min and incubated for 10 min in 3% H_2_O_2,_ washed (3 × 5 min) in PBS (without Ca^++^ and Mg^++^) and then incubated with BSA 3% for blocking background staining. Rabbit monoclonal anti-Ki67 antibody diluted 1∶100 in PBS with 0.05% BSA was applied for 18 h at 4 °C. Sections were than washed (3 × 5 min in PBS, 0.05% BSA) and incubated for 60 min in the appropriate species–specific biotinylated secondary antibodies (goat anti rabbit IgG). Following washings (3 × 5 min in PBS, 0.05% BSA), the sections were incubated for 10 min in streptavidin-conjugated HRP. After this incubation, sections were exposed to 3,3-diaminobenzidine tetrahydrocloride (DAB, detection kit, Millipore, Milan, Italy) for 8 min to produce a brown reaction product. Sections were then counterstained in hematoxylin and mounted in Eukitt® Quick-hardening mounting medium. Only cells with staining of the nuclei were scored as positive. The number of immunoreactive cells was estimated semiquantitatively, as follows: grade +, 10–30% positive cells; grade ++, 30–70% positive cells; and grade +++, > 70% positive cells as described by Bukholm et al. [15].

### Immunofluorescence staining on O.C.T. sections

Seven-μm-thick cryostat sections from tissue samples were used for immunofluorescence staining with anti-CD31 and anti-NG2 antibodies. Sections were rehydrated with PBS and fixed with 4% PFA for 20 min. Subsequently, sections were washed and permeabilized with 0.2% Triton-X100 in PBS for 20 min. After the washes (3 × 5 min) with PBS, sections were blocked with 5% goat serum in PBS. Samples were incubated for 18 h (at 4 °C) with anti-CD31 and anti-NG2 in 5% goat serum in PBS (dilution 1:100). After washes (3 × 5 min) with PBS, secondary antibodies (goat anti-rat Alexa Fluor 568 and goat anti-rabbit Alexa Fluor 488) in 5% goat serum in PBS (dilution 1:200) were applied for 60 min in the dark at room temperature. Samples were washed (3 × 5 min) with PBS and incubated with DAPI in PBS (1:5000). Sections were washed (3 × 5 min) with PBS and mounted in Eukitt® Quick-hardening mounting medium. Quantification of human CD31 was done counting 5 random fields for section, each slide having five sections (10 x magnification) as reported [14].

### Proliferation of HUVEC in co-culture with MCF-7 cells

The co-cultivation transwell apparatus involves the reconstruction of the tumor microenvironment with endothelial and tumor cells separated by polycarbonate membrane to evaluate cell-cell interactions. Co-cultivation models were set up as follows. HUVEC (5 × 10^3^ cells) were plated on the bottom of 24 multiplates pre-coated with gelatin. Tumor cells were seeded at density of 2 × 10^4^ on the top of polycarbonate membrane with 0.4 μm pores. After 24 h incubation for cells adherence, transwells were put in the same 24 multiplates for 48 h of co-culture in EBM medium (without growth factors) additioned with 1% FBS. Bevacizumab was added at 100 ng/ml, where appropriate. Cells were then fixed, stained and randomly counted at 20 x original magnification in 5 fields as previously reported [16].

### Scratch assay in HUVEC co-cultured with MCF-7 cells

Co-cultivation models were set up as follows. HUVEC (1 × 10^5^ cell) were seeded on the bottom of 12 well multiplates pre-coated with gelatin. Tumor cells were seeded at density of 3 × 10^4^ on the top of polycarbonate membrane with 0.4 μm pores. Once HUVEC reached the confluence, cells were scratched using a sterile 100–1000 μl micropipette tip to create a wound ±500 μm across the monolayer and transwells were put in the same 12 multiplates for 18 h of co-culture in EBM medium (without growth factors, but with 1% FBS). The antimitotic ARA-C (2.5 μg/ml) was added in all the transwells, while Bevacizumab (100 ng/ml), where appropriate.

Images of the wound in each well were acquired from 0 to 18 h under a phase contrast microscope (Nikon Eclipse TE 300, Nikon, Tokyo, Japan), at 20 x magnification. The rate of migration was measured by quantifying the uncovered area of wound that HUVEC covered starting from the edge of the scratch. Results are expressed as percentage of area of wound [17].

### Tube formation assay by HUVEC co-cultured with MCF-7 cells

Tumor cells (3 × 10^4^ cells) were cultured on transwell inserts (12 mm diameter, polycarbonate membranes with 0.4 μm pores; Corning, Lowell, MA, USA). After 24 h the inserts were transferred on top of endothelial cells plated on Matrigel (1.5 × 10^5^ cells in 12 well multiplate). After 18 h of incubation, endothelial cells were photographed and network formation on Matrigel was measured by means of the number of branching points (Nikon Eclipse E400 and camera Nikon DS-5MC).

### Permeability assay in HUVEC co-cultured with MCF-7 cells

Permeability assay was performed as previously described [10]. Briefly, MCF-7 (Scr, ALDH1A1KD and ALDH1A1^+^) were seeded at density of 3 × 10^4^ on the bottom of 12 well multiplates. HUVEC (8 × 10^4^ cell) were seeded on the top of polycarbonate membrane with 0.4 μm pores, pre-coated with gelatin. After 24 h incubation necessary for cell adherence, transwells were put in the same 12 multiplates with medium additioned with 1% FBS until HUVEC confluence. Bevacizumab was added, where indicated, at 100 ng/ml. Fluorescein isothiocyanate-dextran (FITC-Dextran) (3 kDa, 10 μM) was used as a fluorescent marker of paracellular permeability, which was evaluated after 15 min by measuring the fluorescence in a plate reader (Infinite 200 Pro, SpectraFluor) at 485 and 535 nm excitation and emission, respectively. Data are reported as fluorescence units, taking as reference MCF-7 in control condition with medium supplemented with 1% serum (without Bevacizumab)*.*

### Immunofluorescence analysis in HUVEC co-cultured with MCF-7 cells

The tight junction protein VE-Cadherin, expressed at the cell surface, was monitored by confocal analysis. A total of 5 × 10^4^ HUVEC were seeded on 1-cm circular glass coverslips added in the bottom of a 12 well multiplate. MCF-7 (Scr, ALDH1A1KD and ALDH1A1^+^) were seeded at density of 3 × 10^4^ on the top of 0.4 μm pore polycarbonate membrane. After 24 h incubation, transwells were put in the same 12 multiplates until HUVEC confluence (in medium with 1% FBS) and Bevacizumab (100 ng/ml) was added where appropriate. Immunofluorescence analysis was performed on endothelial cells as previously reported [18].

### Data analysis and statistical procedures

Results are either representative or the average of at least 3 independent experiments done in triplicate. Statistical analysis was performed using ANOVA test followed by the Bonferroni test and the Student t test when appropriate (GraphPad). *p* < 0.05 was considered statistically significant.

## Results

### Expression and activity of ALDH1A1 isoform in breast cancer cells

**Fig. 1:**
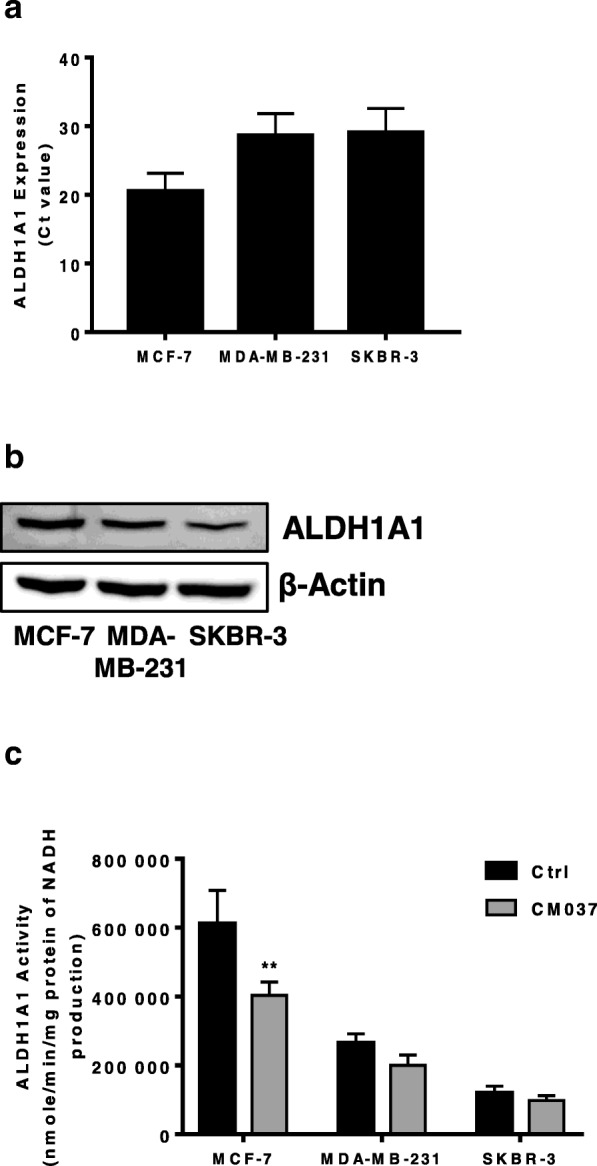
Expression and activity of ALDH1A1 in breast cancer cells. **a** RT-PCR analysis of ALDH1A1 in breast cancer cells grown with 10% FBS. **b** Western blot analysis for ALDH1A1. β-Actin was used as loading control. Gel shown is representative of three experiments with similar results. **c** Variation of ALDH1A1 activity, measured by the formation of NADH in tumor cells. Breast tumor cell lysates were pretreated with CM037 (50 μM, 10 min) and absorbance at 340 nm (corresponding to NADH production) was measured. **p < 0.01 vs untreated cells

We examined ALDH1A1 expression and activity in a panel of cell lines representing different breast cancer histotypes, namely MCF-7, MDA-MB-231 and SKBR-3. We detected ALDH1A1 as mRNA and as protein, in all cell lines (Fig. 1a and b, respectively). The enzyme was metabolically functional as application of selective inhibitor of ALDH1A1 isoform, CM037 (50 μM) [19], produced a variable decline of enzyme activity, maximal inhibition being 40% for MCF-7 (Fig. 1c). Among all cell lines, MCF-7 showed highest expression and activity of ALDH1A1.

### ALDH1A1 activity in MCF-7 cells modulates tumor stemness in vitro

**Fig. 2:**
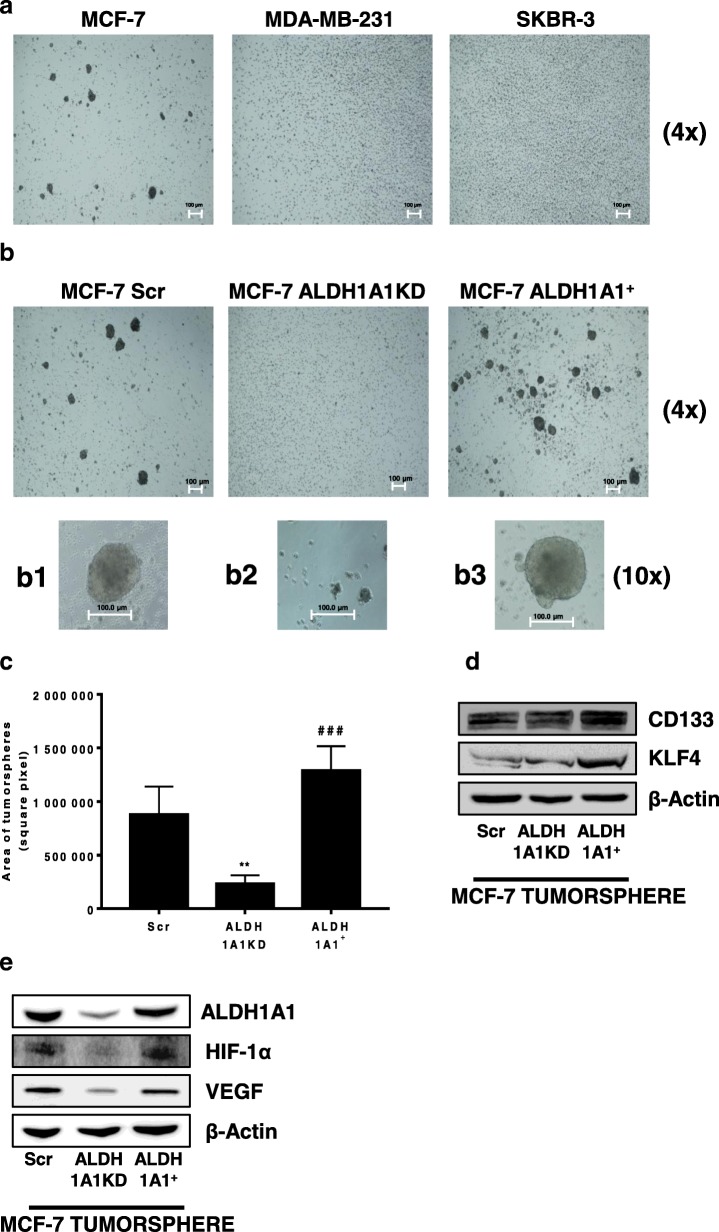
MCF-7 ALDH1A1 affects in vitro stemness. **a** Representative images of tumorspheres (4x magnification) showing morphology of spheroids grown on ultra-low attachment plate. Scale bar, 100 μm. **b** Representative images of tumorspheres (4x magnification) of MCF-7 Scr, MCF-7 ALDH1A1KD and MCF-7 ALDH1A1^+^, showing morphology of spheroids grown on ultra-low attachment plate. Scale bar, 100 μm. **b1, b2, b3.** Representative images of tumorspheres (10x magnification) of MCF-1 Scr, MCF-7 ALDH1A1KD and MCF-7 ALDH1A1^+^, showing morphology of spheroids grown on ultra-low attachment plate. Scale bar, 100 μm. **c** Quantification of MCF-7 tumorspheres. Tumorspheres area were calculated using ImageJ Software. Ten pictures for each well were quantified. Tumorspheres> 10.000 pixel square were considered. **p < 0.01 vs MCF-7 Scr. ###*p* < 0.001 vs MCF-7 ALDH1A1KD. (*n* = 3). **d** Western blot analysis of stemness markers CD133 and KLF4 in MCF-7 Scr, MCF-7 ALDH1A1KD, and MCF-7 ALDH1A1^+^ tumorspheres. **e** Western blot analysis of ALDH1A1, HIF-1α and VEGF in MCF-7 Scr, MCF-7 ALDH1A1KD and MCF-7 ALDH1A1^+^ tumorspheres

To evaluate the association between ALDH1A1 expression/activity and stemness state of the above breast tumor cells, we explored their ability to form tumorpheres in vitro. Among all cell lines, only MCF-7 displayed the ability to form tumorspheres (Fig. 2a). This is consistent with the higher enzymatic activity in these cells. To study whether the activity of ALDH1A1 contributed to mediate the MCF-7 cancer stem-like features, we used loss-of-function and gain-of-function approaches. For loss-of-function model, MCF-7 cells were appropriately transfected as follows: two knockdown clones of ALDH1A1KD (shA and shB), and a scrambled sequence (Scr). Each new cell line was validated by RT-PCR and western blotting which provided the expected results: reduced enzyme mRNA levels and protein expression in MCF-7 ALDH1A1KD (Additional file 2: Figure S1a, S1b). In addition, MCF-7 ALDH1A1KD show an impaired enzyme function evaluated by ALDH1A1 enzymatic activity assay (Additional file 2: Figure S1e). For gain-of-function model, we obtained cells enriched for ALDH1A1 expression, termed ALDH1A1^+^, which displayed an increase in term of expression, as mRNA and protein level, and enzymatic activity (Additional file 2: Figure S1c, S1d, S1e). Under these conditions, the ability to form tumorspheres was drastically impaired in MCF-7 ALDH1A1KD, while it was increased in MCF-7 ALDH1A1^+^ (Fig. 2b, c). We also noted an increased CD133 and KLF4 (stemness markers) expression in ALDH1A1 enriched tumorspheres (Fig. 2d), concomitant with an important decrease of HIF-1α and VEGF expression in MCF-7 ALDH1A1KD cells (Fig. 2e). By contrast, the expression of HIF-1α and VEGF was rescued in tumorspheres of MCF-7 ALDH1A1^+^. All together the results demonstrate that ALDH1A1 activity in MCF-7 breast cancer cells orchestrated both the stemness and the angiogenic output.

### ALDH1A1 activity in MCF-7 cells regulates angiogenic output via retinoic acid signalling

**Fig. 3:**
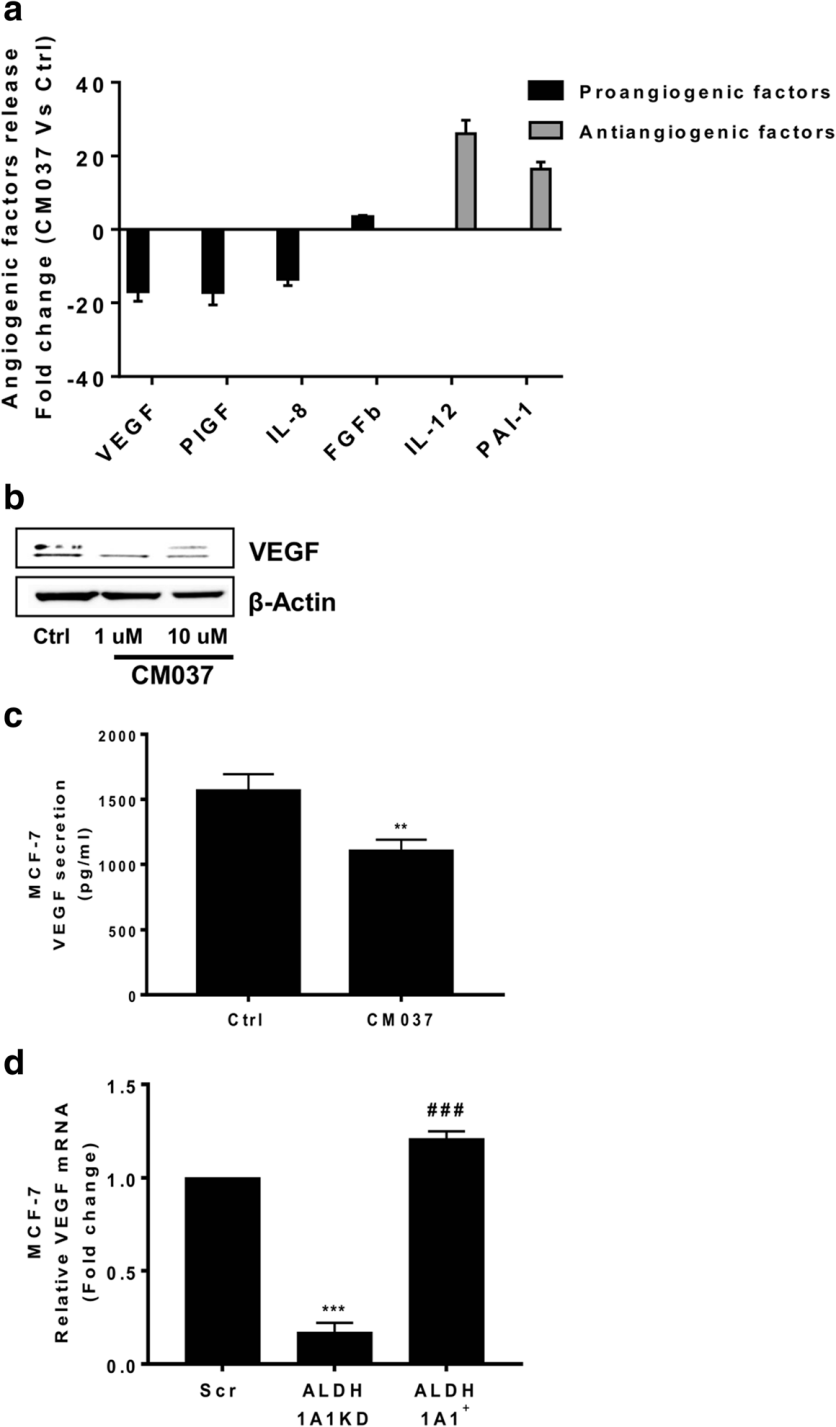
MCF-7 ALDH1A1 regulates angiogenic factor output via retinoic acid signalling. **a** Angiogenic factor release evaluated by ELISA plate array in supernatants of MCF-7 treated with CM037 (1 μM) for 48 h. The experiment was performed 2 times in duplicate. **b** MCF-7 cells were exposed to CM037 at different concentrations (1 and 10 μM) for 18 h and western blot was carried out. β-Actin was used to normalize loading. **c** Cells were treated with CM037 (1 μM, 18 h) and VEGF levels were measured by ELISA assay in MCF-7 conditioned media. After 18 h supernatants were harvested and cells fixed, stained and counted. The number of counted cells was not significantly different. Data are reported as pg/ml. **p < 0.01 vs untreated cells. **d** RT-PCR analysis of VEGF in MCF-7 Scr, MCF-7 ALDH1A1KD and MCF-7 ALDH1A1^+^ cultured in medium with 1% FBS for 48 h. Data are reported as ΔCt (Ct gene of interest-Ct Housekeeping gene). ***p < 0.001 vs MCF-7 Scr. ###*p* < 0.001 vs MCF-7 ALDH1A1KD. **e** Western blot analysis of VEGF and HIF-1α in MCF-7 exposed or not to CoCl_2_ (100 μM, 72 h, 1% FBS). β-Actin was used as loading control. Gel shown is representative of three experiments with similar results. **f** Quantification of blots reported in **e**. *p < 0.05 vs MCF-7 Scr. **p < 0.01 vs MCF-7 Scr. ###*p* < 0.001 vs MCF-7 ALDH1A1KD. **g** Soluble VEGF was detected by ELISA in media conditioned by MCF-7 cells. Cells were seeded in 24-well plates at density 3 × 10^4^ cells/well. After 48 h the supernatants were harvested and cells fixed, stained and counted. The number of counted cells was not significantly different. Data are reported as pg/ml. **p < 0.01 vs MCF-7 Scr. ##*p* < 0.01 vs MCF-7 ALDH1A1KD. **h** HIF-1α and VEGF expression evaluated by western blot in MCF-7 ALDH1A1KD cells exposed for 48 h (1 μM) to exogenous retinoic acid. **i** HIF-1α and VEGF expression in MCF-7 ALDH1A1^+^ treated with RAR antagonist (AGN193109) and RXR antagonist (UVI 3003) for 48 h (each at 1 μM). β-Actin was used as loading control. Gel shown is representative of three experiments with similar results. **j** VEGF and CD133 expression in MCF-7 transiently silenced for HIF-1α. β-Actin was used as loading control. Gel shown is representative of three experiments with similar results

On the basis of the above results, we focused our attention on angiogenic factor output of MCF-7 harbouring different levels of ALDH1A1. First we performed a cytokine ELISA array to profile the expression of key mediators involved in the angiogenesis process. The array was performed on supernatants from MCF-7 cells treated or not with the selective ALDH1A1 blocker, CM037 (Additional file 3: Figure S2a). We found important perturbations in the secretion of angiogenesis inhibitors and inducers. We observed a drastic reduction (> 10-fold) of VEGF release, as well as placental growth factor and IL-8. By contrast, we observed a relevant increase of antiangiogenic factors such as interleukin 12 (IL-12) and plasminogen activator inhibitor, type I (PAI-1). FGF-2 did not significantly change (Fig. 3a and Additional file 4: Table S2). Due to the crucial role of VEGF in breast tumor angiogenesis [20], we examined the expression and release of the growth factor in MCF-7 in which ALDH1A1 activity was impaired. Blockade of ALDH1A1 enzymatic activity with CM037 produced a significant reduction of VEGF expression and release, when compared with naïve MCF-7 (Fig. 3b, c), supporting the contribution of the enzyme activity to MCF-7 angiogenic phenotype. Consistently, in MCF-7 cells harbouring different levels of ALDH1A1, we found that high ALDH1A1 expression was associated with significant increase of VEGF compared to MCF-7 ALDH1A1KD, in terms of mRNA, protein, as well as soluble VEGF levels (Fig. 3d, e, f, g). We obtained similar results in MCF-7 transiently silenced for ALDH1A1 (Additional file 3: Figure S2b). Since VEGF transcription is induced by HIF-1α in tumor cells [21], we also sought to determine whether ALDH1A1 regulates HIF-1α transcriptional activation in MCF-7 cells. In normoxia, HIF-1α was detectable by western blot only in MCF-7 cells enriched of ALDH1A1 (Fig. 3e, f). Using CoCl_2_ to mimic hypoxia condition, we found a variable activation of HIF-1α in MCF-7 cells expressing different levels of ALDH1A1. We observed a greater HIF-1α induction in MCF-7 Scr compared to MCF-7 ALDH1A1KD cells, while in MCF-7 ALDH1A1^+^ cells we detected the highest expression of both HIF-1α and VEGF (Fig. 3e, f).

ALDH1A1 catalyzes the oxidation of retinaldehyde to retinoic acid (RA) [22]. Here, we investigated whether RA signalling might mediate the observed VEGF and HIF-1α regulation in MCF-7 ALDH1A1^+^. Exposure of MCF-7 ALDH1A1KD to exogenous RA (1 μM) restored VEGF and HIF-1α expression (Fig. 3h). In a complementary approach, in MCF-7 ALDH1A1^+^ we found a significant reduction of HIF-1α and VEGF expression in the presence of a RAR blocker (AGN 193109), but not in the presence of UVI3003, a RXR antagonist (Fig. 3i). Finally, we investigated the role of HIF-1α in VEGF expression regulation. In MCF-7 ALDH1A1^+^ cells the silencing of HIF-1α (by siRNA) reduced VEGF expression. In addition we found a reduction of CD133 expression, suggesting a role of HIF-1α in the maintenance of the stem-like phenotype (Fig. 3j).

These data indicate a contribution of ALDH1A1 activity on HIF-1α and VEGF expression in MCF-7 cells which is mediated through retinoic acid signalling, and in particular through the selective RAR pathway.

### ALDH1A1 regulates angiogenesis in a VEGF-dependent manner

**Fig. 4:**
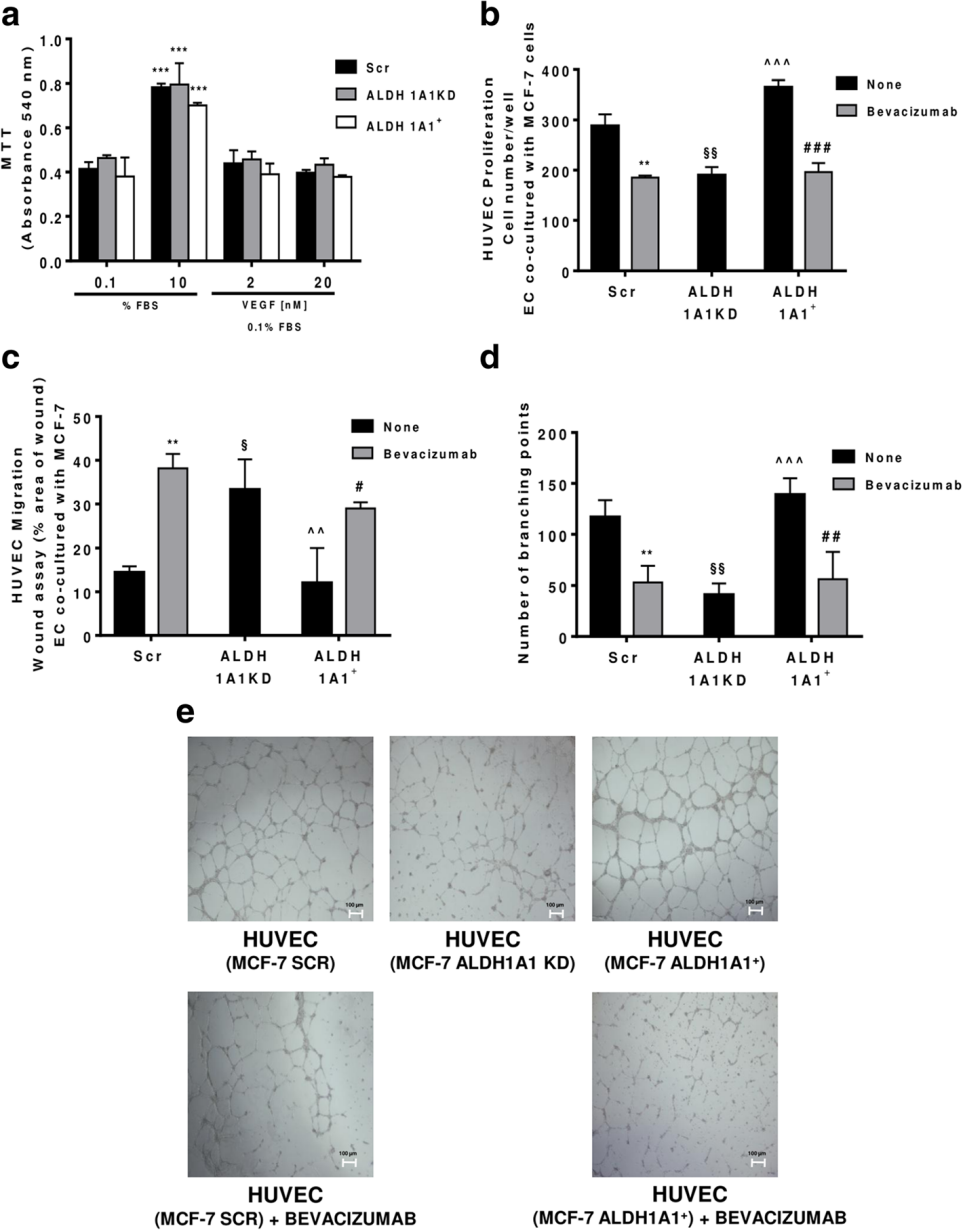
MCF-7 ALDH1A1 regulates endothelial angiogenic features in VEGF dependent manner. **a** Viability of MCF-7 (Scr, ALDH1A1KD, ALDH1A1^+^) exposed to exogenous serum (10% FBS) or VEGF (2 and 20 ng/ml) at 72 h and evaluated by MTT assay. Data are reported as absorbance at 540 nm. ***p < 0.001 vs 0.1% FBS group. **b** MCF-7 were co-cultured with HUVEC for 48 h (1% FBS) in presence of Bevacizumab (100 ng/ml); HUVEC were fixed, stained and counted (5 fields random for well). Data are reported as number of HUVEC counted/well. (*n* = 3). **p < 0.01 vs HUVEC co-cultured with MCF-7 Scr without Bevacizumab. ###*p* < 0.001 vs HUVEC co-cultured with MCF-7 ALDH1A1^+^ without Bevacizumab. §§*p* < 0.01 vs HUVEC co-cultured with MCF-7 Scr without Bevacizumab. ^^^*p* < 0.001 vs HUVEC co-cultured with MCF-7 ALDH1A1KD. **c** Tumor cells were co-cultured with MCF-7 for 18 h (1% FBS) in presence of Bevacizumab (100 ng/ml). Data are reported as % area of migration ratio (% of area at 18 h/area at 0 h). **p < 0.01 vs HUVEC co-cultured with MCF-7 Scr without Bevacizumab. #*p* < 0.05 vs MCF-7 ALDH1A1^+^ without Bevacizumab. §*p* < 0.05 vs HUVEC co-cultured with MCF-7 Scr without Bevacizumab. ^^*p* < 0.01 vs HUVEC co-cultured with MCF-7 ALDH1A1KD. **d** Quantification of branching points of HUVEC seeded in Matrigel layer and co-cultured MCF-7 for 18 h (1% FBS). The results represent the media of 5 pictures. **p < 0.01 vs HUVEC co-cultured with MCF-7 Scr without Bevacizumab. ##*p* < 0.01 vs MCF-7 ALDH1A1^+^ without Bevacizumab. §§*p* < 0.01 vs HUVEC co-cultured with MCF-7 Scr without Bevacizumab. ^^^*p* < 0.001 vs HUVEC co-cultured with MCF-7 ALDH1A1KD. **e** Representative pictures of HUVEC network (4x magnification). **f** Tumor cells were seeded at the bottom of 12-well plates with HUVEC in transwells. The cells have been maintained in co-culture until HUVEC monolayer formation in presence or not of Bevacizumab (100 ng/ml). (*n* = 3). *p < 0.05 vs HUVEC co-cultured with MCF-7 Scr without Bevacizumab. ##*p* < 0.01 vs MCF-7 ALDH1A1^+^ without Bevacizumab. §*p* < 0.05 vs HUVEC co-cultured with MCF-7 Scr without Bevacizumab. ^^*p* < 0.01 vs HUVEC co-cultured with MCF-7 ALDH1A1KD. **g** HUVEC were co-cultured with MCF-7 until confluent in presence, or not of Bevacizumab (100 ng/ml). Immunofluorescent images for VE-Cadherin were obtained by confocal microscope (TCS SP5 Leica). Scale bars, 50 μm

To evaluate the biological relevance of tumoral VEGF, we examined MCF-7 viability vis a vis to the exposure to either varying levels of endogenous ALDH1A1 expression, i.e. MCF-7 Scr, ALDH1A1KD and ALDH1A1^+^, or to exogenous VEGF. We failed to observe any significant viability change among the examined cells (Fig. 4a). In view of this, together with the observation that both MCF-7 Scr and MCF-7 ALDH1A1^+^ cells exhibited a large output of VEGF (see Fig. 3d, e, f), we hypothesized that the released VEGF acted solely in a paracrine manner on the surrounding endothelial cells. Consistently, when we examined several aspects of endothelial cell behaviour in co-culture with ALDH1A1KD breast tumor cells in a transwell apparatus, we found that HUVEC proliferation was drastically reduced by co-culture with MCF-7 ALDH1A1KD. By contrast, HUVEC co-cultured with MCF-7 ALDH1A1^+^ showed a significant increase of proliferation (Fig. 4b). By examining HUVEC migration (in vitro scratch assay), we found that MCF-7 Scr and ALDH1A1^+^ provided pro-migratory stimulus (Fig. 4c), while co-incubation with MCF-7 ALDH1A1KD, failed to affect endothelial migration. A co-culture experiment between HUVEC and any of the above mentioned MCF-7, verified the hypothesis as it showed large increases of both proliferation and migration of HUVEC co-incubated with MCF-7 cells, except for ALDH1A1KD. Addition of Bevacizumab, the known VEGF blocking antibody, to the co-culture blunted these responses in terms of HUVEC proliferation and migration (Fig. 4b, c).

To strengthen the above results, we investigated tube formation by ECs seeded on Matrigel layers. In co-culture with MCF-7 ALDH1A1^+^ cells, HUVEC formed net-like structures, which were impaired by Bevacizumab addition (Fig. 4d, e). Conversely, endothelial cells co-cultured with MCF-7 ALDH1A1KD showed a slight ability to form tubes (Fig. 4d, e).

Similar results were obtained by measuring endothelial permeability. HUVEC cells co-cultured with MCF-7 ALDH1A1^+^ and MCF-7 Scr cells were more permeable compared to HUVEC co-cultured with MCF-7 ALDH1A1KD cells (Fig. 4f). Increased permeability was associated with a fading of VE-Cadherin, a cell-cell contact protein involved in tight junction signaling, evaluated by immunofluorescence (Fig. 4g).

Collectively, these findings suggest the existence of a dynamic cross talk between tumor breast cells and the endothelium, favored by tumor ALDH1A1 expression which facilitates endothelial cell recruitment in a VEGF-dependent manner.

### ALDH1A1 mediates tumor growth and angiogenesis in vivo

**Fig. 5:**
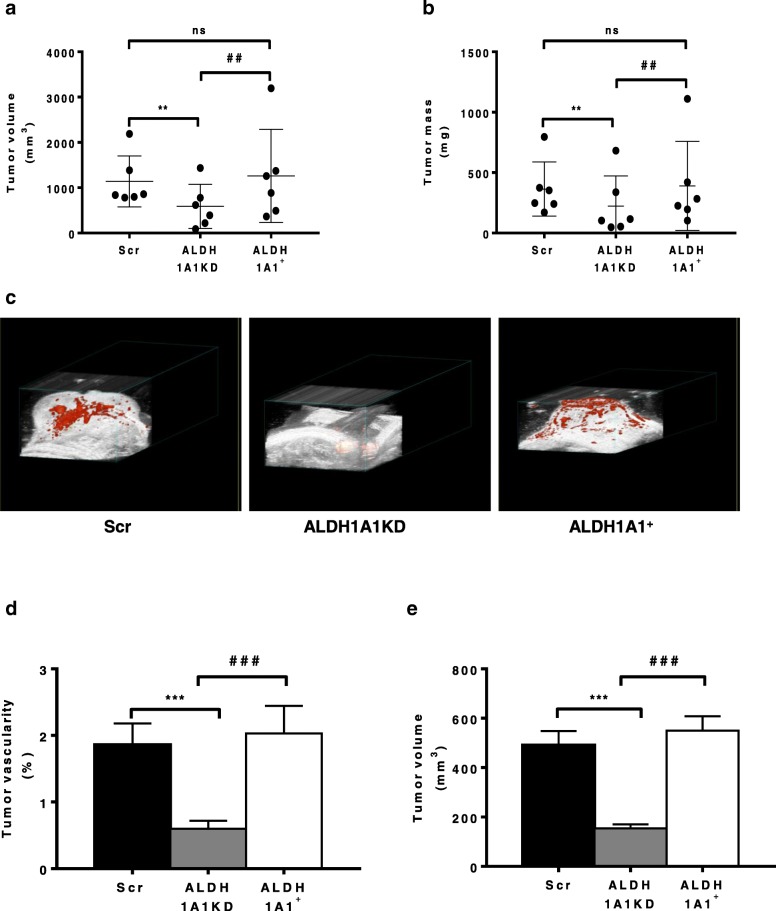
ALDH1A1 affects tumor growth and vascular flow in MCF-7 tumor xenograft in athymic nude mice. MCF-7 cells (1 × 10^7^ with 50% *v*/v of Matrigel) were injected s.c. in flank of athymic female nude mice. β-estradiol were injected (3 mg/kg), every 7 days i.m.. All mice were sacrificed at day 23. Tumor volumes were detected twice a week using a caliper and calculated by the formula: shortest diameter × longest diameter × thickness of the tumor in mm (*n* = 6 animals per group). **a** Tumor volume at day 23. **p < 0.01 vs Scr group. ##*p* < 0.01 vs ALDH1A1^+^ group. **b** Tumor mass at day 23. The tumors were weighted immediately after isolation from mice. **p < 0.01 vs Scr group. ##*p* < 0.01 vs ALDH1A1^+^ group. **c** Power Doppler imaging in tumors using 3D Power Doppler imaging VisualsonicVevo 2100 at the day of sacrifice. The tumor volume and percent vascularity are calculated. Red areas indicate blood flow. Images are representative of six mice per group**. d** Quantification of tumor vascularity (as %) by VisualsonicVevo 2100 before the sacrifice. ***p < 0.001 vs Scr group. ###*p* < 0.001 vs ALDH1A1^+^ group. **e** Quantification of tumor volumes by VisualsonicVevo 2100 at the day of sacrifice. ***p < 0.001 vs Scr group. ###*p* < 0.001 vs ALDH1A1^+^ group

**Fig. 6:**
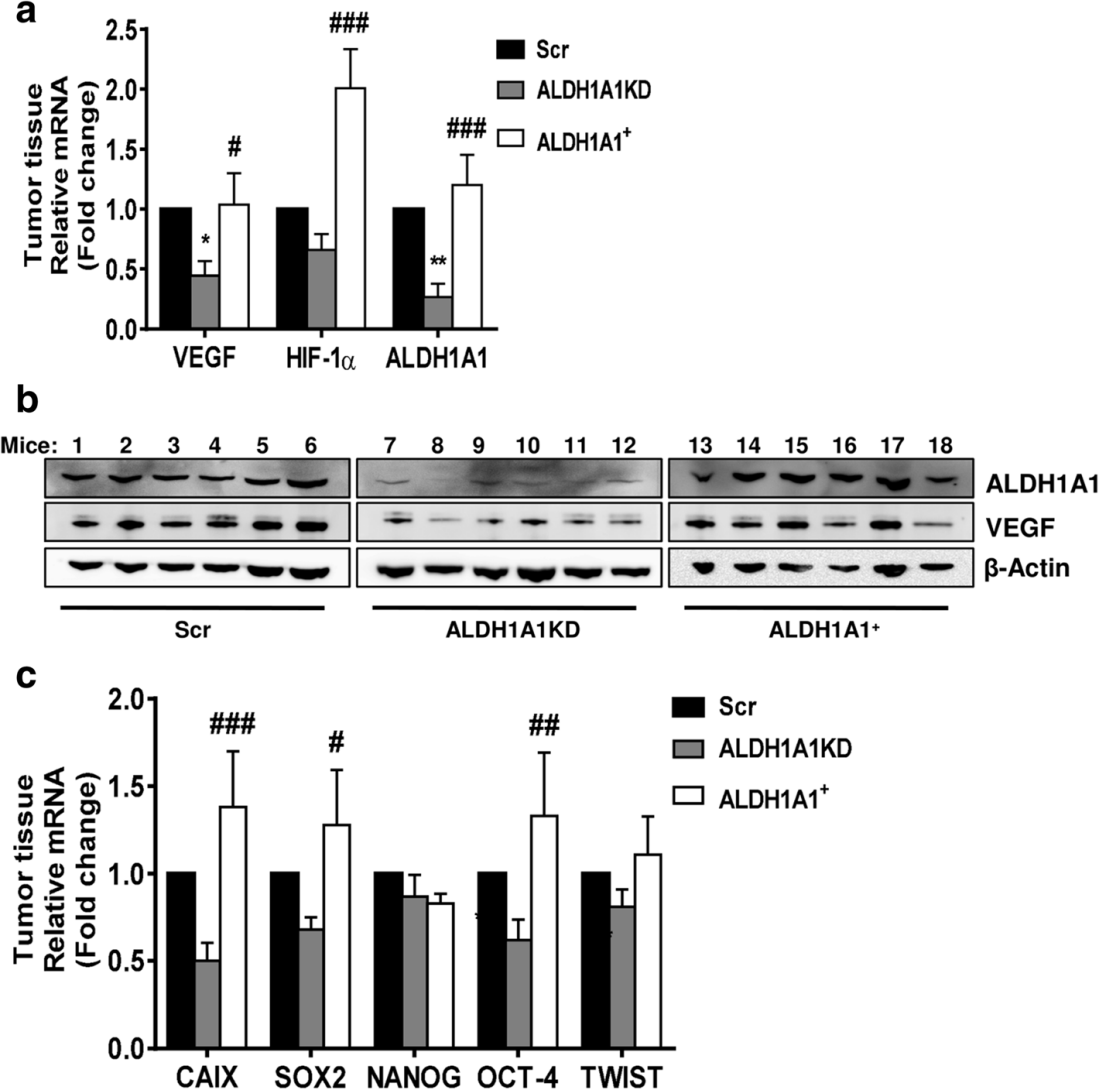
ALDH1A1 influences tumor angiogenesis and VEGF production in vivo. **a** Evaluation of VEGF, HIF-1α and ALDH1A1 RNA in tumor samples. Frozen tumors were homogenized and RNA was extracted to perform RT-PCR analysis of VEGF, HIF-1α and ALDH1A1 mRNA. Data are reported as ΔCt (Ct gene of interest-Ct Housekeeping gene). Each bar is the mean of 6 different tumors. The experiment was repeated two times. *p < 0.05 vs Scr group. **p < 0.01 vs Scr group. #*p* < 0.05 vs ALDH1A1KD group. ###*p* < 0.001 vs ALDH1A1KD group. **b** Evaluation of VEGF and ALDH1A1 proteins in tumor samples. Tissues were harvested, homogenized and sonicated. Subsequently, proteins were extracted and western blot was performed. β-Actin was used as loading control. The experiment was repeated two times. **c** Evaluation of mRNA for CAIX (HIF-1α target gene) and stemness markers (SOX2, NANOG, OCT-4 and TWIST) in tumor samples. Each bar is the mean of 6 different tumors. The experiment was repeated two times. #*p* < 0.05 vs ALDH1A1KD group. ##*p* < 0.01 vs ALDH1A1KD group. ###*p* < 0.001 vs ALDH1A1KD group. **d** Evaluation of HIF-1α and stemness markers (CD133, KLF4 and SOX2) proteins in tumor samples. The experiment was repeated two times. **e** Quantification of blots reported in **d**. *p < 0.05 vs Scr group. #*p* < 0.05 vs ALDH1A1KD group. ##*p* < 0.01 vs ALDH1A1KD group. **f** Quantification of microvessel density by human CD31 staining (magnification 20x) was done counting 5 random fields for section, each slide having five sections. **p < 0.01 vs Scr group. ##*p* < 0.01 vs ALDH1A1^+^ group. **g** Representative images of immunostaining for CD31 (red) and DAPI (blue) in tumor sections from Scr (left), ALDH1A1KD (center) or ALDH1A1^+^ (right) mice. Pictures report different vessel densities in tumors. Magnification 20x. Scale bar, 50 μm. **h** Representative images of double-immunostaining for CD31 (red) and NG2 (green) in tumor sections from Scr (left), ALDH1A1KD (center) or ALDH1A1^+^ (right) mice. DAPI staining is blue. Magnification 40x. Scale bars, 50 μm

Next, we determined whether the reduced expression and activity of ALDH1A1 in breast cancer cells might influence in vivo tumor angiogenesis. We implanted s.c. in nude mice MCF-7 ALDH1A1KD, MCF-7 ALDH1A1^+^ and Scr and monitored tumor growth up to 23 days after inoculation. In mice bearing MCF-7 ALDH1A1KD cells, tumor growth and mass were significantly reduced, compared to MCF-7 Scr and MCF-7 ALDH1A1^+^ (Fig. 5a, b). In the MCF-7 ALDH1A1KD tumors, Ki67 staining was detected in 10–30% of tumor cells (+), whereas it was 70% in ALDH1A1^+^ or Scr tumors (+++) (Additional file 5: Figure S3) indicating that high proliferative activity was associated with ALDH1A1 expression in tumors. Further, to study ALDH1A1 involvement in tumor angiogenesis, we examined tumor blood supply, monitoring the vascularity of the entire tumor volume by 3D Power Doppler imaging Visualsonic Vevo 2100. The tumor vascularity, expressed as % of total, was highest in ALDH1A1^+^ and Scr specimen (2.032 vs 1.862%, respectively), while it was drastically reduced in ALDH1A1KD (0.599%, Fig. 5c, d and e). Evaluation of VEGF expression as mRNA and protein in tumor explants (Fig. 6a, b), revealed its highest levels in ALDH1A1^+^ and Scr tumor specimen, whereas it was strongly downregulated in ALDH1A1KD tumors. Further, the expression of HIF-1α and CAIX (HIF-1α target gene) mRNA and HIF-1α protein were significantly increased in ALDH1A1^+^ compared to Scr and ALDH1A1KD specimen (Fig. 6a, c, d, e). Finally, the expression of stemness markers SOX2 and OCT-4 was increased as mRNA level in tumor enriched with ALDH1A1 (Fig. 6c). At protein level, we found an increase of CD133, SOX2 and KLF4 expression in the same tumors (Fig. 6d, e).

Together these findings suggest the existence of a close relationship between ALDH1A1 activity, HIF-1α and VEGF expression. Because a number of reports have described a causal relationship between VEGF levels and extent of MVD in breast cancer [23–26], we thought of interest to determine the density of angiogenic microcapillaries in MCF-7 tumors. By immunostaining for CD31, we found a reduced density of vessels in ALDH1A1KD tumors compared to ALDH1A1^+^ ones (Fig. 6f, g). Double immunostaining for CD31 and NG2 (marker of pericytes) evidences that in ALDH1A1KD tumor vessels had aberrant morphology and appeared tortuous, leaky and much less structured (Fig. 6h).

Taken together these results show that the level of ALDH1A1 correlates with angiogenic phenotype, increased HIF-1α, VEGF, and MVD in tumors.

## Discussion

ALDH1A1, a cytosolic enzyme expressed in several solid tumors [22], is considered a marker of a subset of cancer stem cells endowed with aggressive traits. CSCs have been reported to drive tumor progression and metastasis formation, and their activation is associated with poor prognosis, particularly in breast cancer [27]. The mechanism underlying tumor aggressiveness promoted by ALDH1A1 expression in certain breast cancer phenotypes remains poorly defined. Here we describe a mechanism, focused on ALDH1A1/HIF-1α/VEGF axis activation through retinoic acid signalling. The evidence gathered in this work shows that ALDH1A1 promotes robust angiogenesis in vitro and in vivo in breast cancer cell lines inoculated in mice, by inducing VEGF expression and release, as a consequence of upstream HIF-1α activation. The stimulation appears closely associated with the upregulation of ALDH1A1 as it was observed only in MCF-7 cells, particularly in those in which the enzyme was overexpressed (MCF-7 ALDH1A1^+^). These cells, possessing an increase of ALDH1A1 activity, display a number of features such as enhanced capability to form tumorspheres, an indication for the formation of a cancer stem cell niche within the tumor. Importantly, the high ALDH1A1 expression confers to MCF-7 a proangiogenic phenotype mediated by increased VEGF expression and release, and in endothelial cells co-cultured with MCF-7 tumor cells, the release of VEGF induces an angiogenic response, measured as endothelial proliferation, migration, tube formation and permeability. Moreover, exposure to the enzyme inhibitor CM037 significantly reduced VEGF expression and release, suggesting that the level of ALDH1A1 expression and activity is critical for the development of an aggressive tumor phenotype. An interesting finding of this work relates to the role of tumor-released VEGF on MCF-7 proliferation, as it shows that the growth factor produced by tumor cells elicits proangiogenic functions in endothelial cells, whereas it fails to stimulate tumor cell changes. These data are in agreement with data from the literature, reporting that MCF-7 express low levels of VEGF receptors [28]. Further insight on the ALDH1A1 mechanism as a driver of tumor progression was gained by examining two signalling pathways, i.e. retinoic acid and HIF-1α. HIF-1α is a key transcriptional factor for angiogenesis and metabolic reprogramming of tumor cells [29], and it is known that retinoic acid induces VEGF and HIF-1α in some tumors cells and promotes breast tumor progression depending on the cellular context [9, 30–32]. VEGF may be transcriptionally regulated by other molecular mechanisms in response to extracellular stimuli [30, 33]. A contribution of HIF-1α and additional pathways may explain the modulation of VEGF in ALDH-dependent manner.

Indeed, the findings that ALDH1A1 enriched cells (MCF-7 ALDH1A1^+^) show a higher HIF-1α expression level, that upon retinoic acid exposure restores the VEGF decline observed in ALDH1A1 silenced cells (MCF-7 ALDH1A1KD), suggest a potential interwoven loop of these three signalling molecules. Further, enhancement of HIF-1α observed in MCF-7 ALDH1A1^+^ is consistent with the higher tumor growth by the reprogramming of metabolism of tumor cells toward an oxygen-independent biochemical pathway [34] and the sustainment of stemness property in many cancers [35]. The interconnection between the stem properties and metabolism in tumor cells has recently defined metabostemness [36].

Finally, in vivo experiments on nude mice comparing the vascularity development as well as other changes (VEGF intratumoral levels, tumor mass and Ki67 index) following s.c. inoculation of Scr, or MCF-7 ALDH1A1^+^ or MCF-7 ALDH1A1KD tumor cells, provided results comparable to those obtained in vitro. In fact, we observed very significant differences between the above groups, with the former ones showing the highest whole tumor vascularity, microvascular density, tumor volume, immunostaining for CD31, and ALDH1A1 mRNA expression levels. In contrast, in cells silenced for ALDH1A1 (MCF-7 ALDH1A1KD) we noted a distinct decline of the above parameters.

## Conclusion

CSCs are able to remodel the tumor microenvironment to promote tumor progression, survival and chemoresistance [37]. Knowledge about interaction between CSCs and their microenvironment is important in developing of new treatment strategy [38] and eradication of CSCs can represent the key to success of cancer treatment [39]. Our results, collectively, suggest a close relationship between ALDH1A1 expression levels and the aggressive traits manifested in MCF-7 breast tumor cells, which appears to be predominantly driven by enhanced angiogenesis.

Taken together, these data identify target molecules which might allow to develop potential therapeutic interventions for harnessing the malicious loop of MCF-7 breast cancer.

## Additional file

Additional file 1: **Table S1.** List of qPCR primers. (PDF 142 kb)

Additional file 2: **Figure S1.** Loss-of function and gain-of function validation to study ALDH1A1 in MCF-7 cells. **a**. RT-PCR in breast tumor cells (Src and ALDH1A1KD, clones shA and shB) cultured in 10 % FBS for 48 h. ***p < 0.001 vs Scr cells. **b**. Western blot analysis of breast tumor cells (Src and ALDH1A1KD, clones shA and shB) cultured in 10 % FBS for 48 h. **c**. RT-PCR analysis of MCF-7 (Src, ALDH1A1KD and ALDH1A1+) cultured in 10 % FBS for 48 h. ***p < 0.001 vs MCF-7 Scr. ^###^*p* < 0.001 vs MCF-7 ALDH1A1KD (*n* = 3). **d**. Western blot analysis of MCF-7 (Src, ALDH1A1KD and ALDH1A1+) cultured in 10 % FBS for 48 h. β-actin was used as loading control. Gel shown is representative of three experiments with similar results. **e**. Enzymatic activity in MCF-7 ALDH1A1KD and ALDH1A1+ evaluated by NADH production. Data are reports as in Figure 1. ***p < 0.001 vs MCF-7 Scr. ^###^p < 0.001 vs MCF-7 ALDH1A1KD. (PDF 372 kb)

Additional file 3: **Figure S2.** ALDH1A1 activity promotes the release of angiogenic factors in MCF-7. **a**. Cytokine ELISA plate array in supernatants of MCF-7 treated with CM037 (1 μM) for 48 h. **b**. Western blot analysis for ALDH1A1 and VEGF on MCF-7 transiently silenced for ALDH1A1 (two sequences of SiRNA, A and B). (PDF 363 kb)

Additional file 4: **Table S2.** Angiogenic factors release evaluated by ELISA plate array in supernatants of MCF-7 treated with CM037 (1 μM) for 48 h. (PDF 71 kb)

Additional file 5: **Figure S3.** Ki67 index is associated with ALDH1A1 expression in mice tumors. Representative images of immunostaining for Ki67. The number of immunoreactive cells was estimated semi-quantitatively. Tumors ALDH1A1+ and Scr had greater 70 % of positive cells and were scored as +++. Tumors ALDH1A1KD had 10-30 % of positive cells and were scored as +. Magnification 20x. Scale bar, 50 μm. (PDF 298 kb)

## Abbreviations

ALDH1A1: Aldehyde dehydrogenase 1A1
CSC: Cancer stem cells
ECs: Endothelial cells
FGF-2: Fibroblast growth factor-2
HIF-1α: Hypoxia inducible factor 1-α
HUVEC: Human umbilical vein endothelial cells
IL-8: Interleukin 8
MVD: Microvessel density
RA: Retinoic acid
RAR: Retinoic acid receptor
RAREs: Retinoic acid responsive elements
VEGF: Vascular endothelial growth factor
