# Correction to: Stemness marker ALDH1A1 promotes tumor angiogenesis via retinoic acid/HIF-1α/VEGF signalling in MCF-7 breast cancer cells

**DOI:** 10.1186/s13046-019-1045-y

**Published:** 2019-02-01

**Authors:** Valerio Ciccone, Erika Terzuoli, Sandra Donnini, Antonio Giachetti, Lucia Morbidelli, Marina Ziche

**Affiliations:** 1 Department of Life Sciences, University of Siena, Via A. Moro 2, 53100 Siena, Italy; 2 Department of Medicine, Surgery and Neuroscience, University of Siena, Via A. Moro 2, 53100 Siena, Italy

## Correction

In the publication of this article [1], there are errors in Figs. 3, 4 and 6. This has now been updated in the original article [1]. The authors declare that the correction does not change the results or conclusions of this paper.

**Fig. 3:**
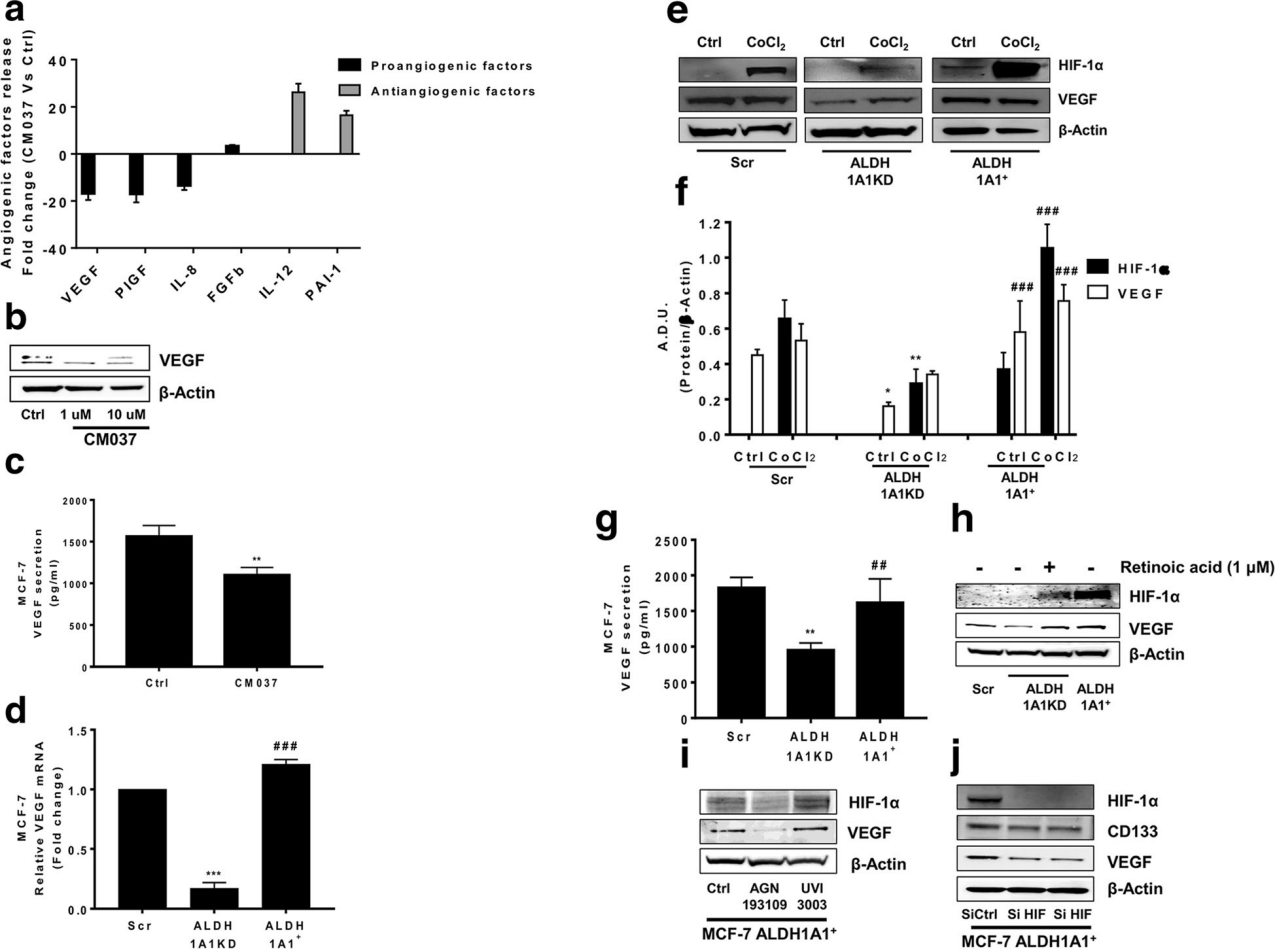
MCF-7 ALDH1A1 regulates angiogenic factor output via retinoic acid signalling. **a** Angiogenic factors release evaluated by ELISA plate array in supernatants of MCF-7 treated with CM037 (1 μM) for 48 h. The experiment was performed 2 times in duplicate. **b** MCF-7 cells were exposed to CM037 at different concentrations (1 and 10 μM) for 18 h and western blot was carried out. β-Actin was used to normalize loading. **c** Cells were treated with CM037 (1 μM, 18 h) and VEGF levels were measured by ELISA assay in MCF-7 conditioned media. After 18 h supernatants were harvested and cells fixed, stained and counted. The number of counted cells was not significantly different. Data are reported as pg/ml. **p < 0.01 vs untreated cells. **d** RT-PCR analysis of VEGF in MCF-7 Scr, MCF-7 ALDH1A1KD and MCF-7 ALDH1A1^+^ cultured in medium with 1% FBS for 48 h. Data are reported as ΔCt (Ct gene of interest-Ct Housekeeping gene). ***p < 0.001 vs MCF-7 Scr. ###p < 0.001 vs MCF-7 ALDH1A1KD. **e** Western blot analysis of VEGF and HIF-1α in MCF-7 exposed or not to CoCl2 (100 μM, 72 h, 1% FBS). β-Actin was used as loading control. Gel shown is representative of three experiments with similar results. **f** Quantification of blots reported in e. *p < 0.05 vs MCF-7 Scr. **p < 0.01 vs MCF-7 Scr. ### *p* < 0.001 vs MCF-7 ALDH1A1KD. **g** Soluble VEGF was detected by ELISA in media conditioned by MCF-7 cells. Cells were seeded in 24-well plates at density 3 × 104 cells/well. After 48 h the supernatants were harvested and cells fixed, stained and counted. The number of counted cells was not significantly different. Data are reported as pg/ml. **p < 0.01 vs MCF-7 Scr. ##*p* < 0.01 vs MCF-7 ALDH1A1KD. **h** HIF-1α and VEGF expression evaluated by western blot in MCF-7 ALDH1A1KD cells exposed for 48 h (1 μM) to exogenous retinoic acid. **i** HIF-1α and VEGF expression in MCF-7 ALDH1A1^+^ treated with RAR antagonist (AGN193109) and RXR antagonist (UVI 3003) for 48 h (each at 1 μM). β-Actin was used as loading control. Gel shown is representative of three experiments with similar results. **j** VEGF and CD133 expression in MCF-7 transiently silenced for HIF-1α. β-Actin was used as loading control. Gel shown is representative of three experiments with similar results

**Fig. 4:**
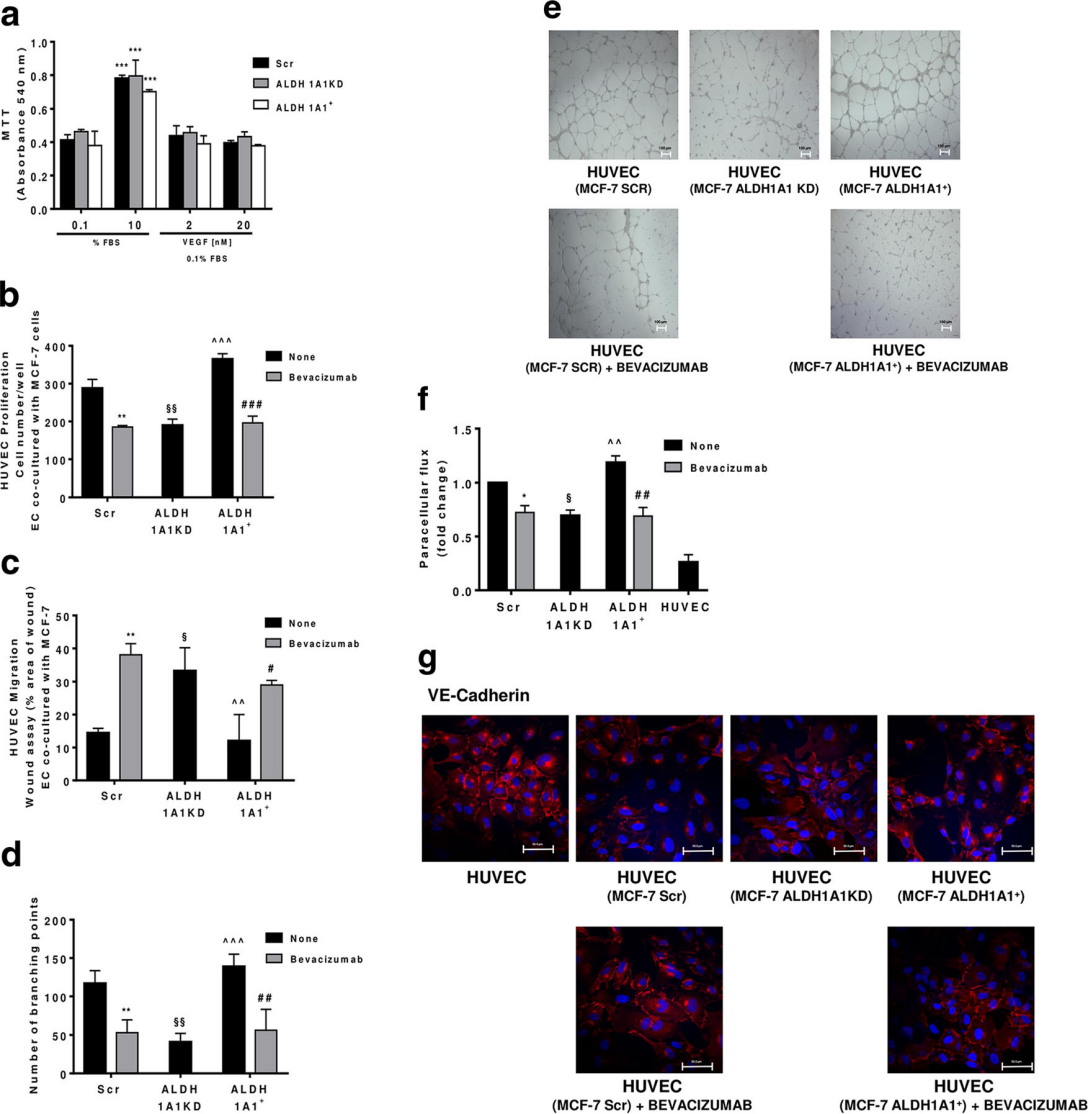
MCF-7 ALDH1A1 regulates endothelial angiogenic features in VEGF dependent manner. **a** Viability of MCF-7 (Scr, ALDH1A1KD, ALDH1A1+) exposed to exogenous serum (10% FBS) or VEGF (2 and 20 ng/ml) at 72 h and evaluated by MTT assay. Data are reported as absorbance at 540 nm. ***p < 0.001 vs 0.1% FBS group. **b** MCF-7 were co-cultured with HUVEC for 48 h (1% FBS) in presence of Bevacizumab (100 ng/ml); HUVEC were fixed, stained and counted (5 fields random for well). Data are reported as number of HUVEC counted/well (*n* = 3). **p < 0.01 vs HUVEC co-cultured with MCF-7 Scr without Bevacizumab. ###*p* < 0.001 vs HUVEC co-cultured with MCF-7 ALDH1A1^+^ without Bevacizumab. §§*p* < 0.01 vs HUVEC co-cultured with MCF-7 Scr without Bevacizumab. ^^^*p* < 0.001 vs HUVEC co-cultured with MCF-7 ALDH1A1KD. **c** Tumor cells were co-cultured with MCF-7 for 18 h (1% FBS) in presence of Bevacizumab (100 ng/ml). Data are reported as % area of migration ratio (% of area at 18 h/area at 0 h). **p < 0.01 vs HUVEC co-cultured with MCF-7 Scr without Bevacizumab. #*p* < 0.05 vs MCF-7 ALDH1A1^+^ without Bevacizumab. §p < 0.05 vs HUVEC co-cultured with MCF-7 Scr without Bevacizumab. ^^*p* < 0.01 vs HUVEC co-cultured with MCF-7 ALDH1A1KD. **d** Quantification of branching points of HUVEC seeded in Matrigel layer and co-cultured MCF-7 for 18 h (1% FBS). The results represent the media of 5 pictures. **p < 0.01 vs HUVEC co-cultured with MCF-7 Scr without Bevacizumab. ##*p* < 0.01 vs MCF-7 ALDH1A1^+^ without Bevacizumab. §§*p* < 0.01 vs HUVEC co-cultured with MCF-7 Scr without Bevacizumab. ^^^*p* < 0.001 vs HUVEC co-cultured with MCF-7 ALDH1A1KD. **e** Representative pictures of HUVEC network (4x magnification). **f** Tumor cells were seeded at the bottom of 12-well plates with HUVEC in transwells. The cells have been maintained in co-culture until HUVEC monolayer formation in presence or not of Bevacizumab (100 ng/ml) (*n* = 3). *p < 0.05 vs HUVEC co-cultured with MCF-7 Scr without Bevacizumab. ##*p* < 0.01 vs MCF-7 ALDH1A1^+^ without Bevacizumab. §*p* < 0.05 vs HUVEC co-cultured with MCF-7 Scr without Bevacizumab. ^^*p* < 0.01 vs HUVEC co-cultured with MCF-7 ALDH1A1KD. **g** HUVEC were co-cultured with MCF-7 until confluent in presence, or not of Bevacizumab (100 ng/ml). Immunofluorescent images for VE-Cadherin were obtained by confocal microscope (TCS SP5 Leica). Scale bars, 50 μm

**Fig. 6:**
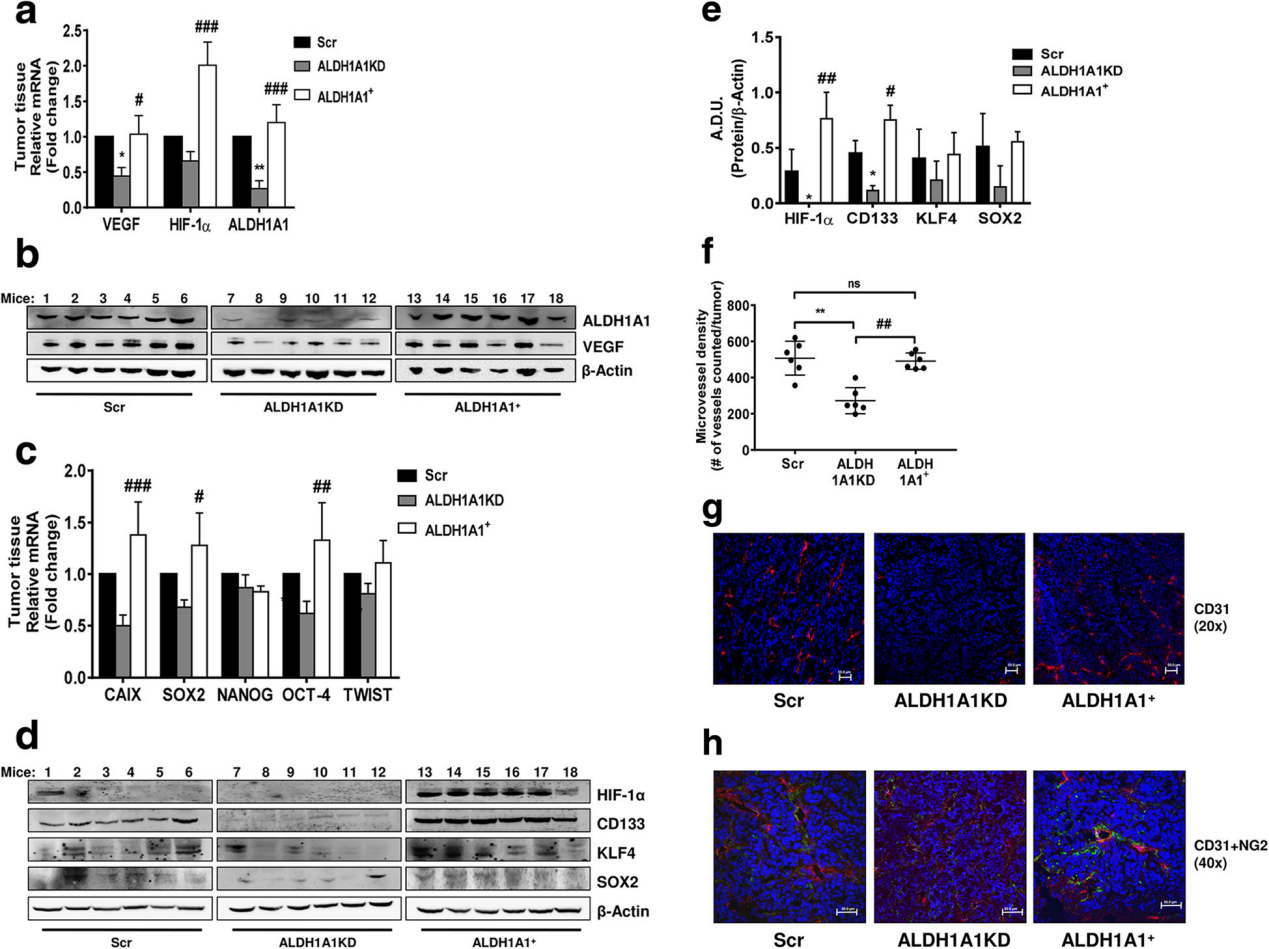
ALDH1A1 influences tumor angiogenesis and VEGF production in vivo. **a** Evaluation of VEGF, HIF-1α and ALDH1A1 RNA in tumor samples. Frozen tumors were homogenized and RNA was extracted to perform RT-PCR analysis of VEGF, HIF-1α and ALDH1A1 mRNA. Data are reported as ΔCt (Ct gene of interest-Ct Housekeeping gene). Each bar is the mean of 6 different tumors. The experiment was repeated two times. *p < 0.05 vs Scr group. **p < 0.01 vs Scr group. #*p* < 0.05 vs ALDH1A1KD group. ###*p* < 0.001 vs ALDH1A1KD group. **b** Evaluation of VEGF and ALDH1A1 proteins in tumor samples. Tissues were harvested, homogenized and sonicated. Subsequently, proteins were extracted and western blot was performed. β-Actin was used as loading control. The experiment was repeated two times. **c** Evaluation of mRNA for CAIX (HIF-1α target gene) and stemness markers (SOX2, NANOG, OCT-4 and TWIST) in tumor samples. Each bar is the mean of 6 different tumors. The experiment was repeated two times. #*p* < 0.05 vs ALDH1A1KD group. ##*p* < 0.01 vs ALDH1A1KD group. ###*p* < 0.001 vs ALDH1A1KD group. **d** Evaluation of HIF-1α and stemness markers (CD133, KLF4 and SOX2) proteins in tumor samples. The experiment was repeated two times. **e** Quantification of blots reported in d. *p < 0.05 vs Scr group. #p < 0.05 vs ALDH1A1KD group. ##*p* < 0.01 vs ALDH1A1KD group. **f** Quantification of microvessel density by human CD31 staining (magnification 20x) was done counting 5 random fields for section, each slide having five sections. **p < 0.01 vs Scr group. ##*p* v 0.01 vs ALDH1A1+ group. **g** Representative images of immunostaining for CD31 (red) and DAPI (blue) in tumor sections from Scr (left), ALDH1A1KD (center) or ALDH1A1^+^ (right) mice. Pictures report different vessel densities in tumors. Magnification 20x. Scale bar, 50 μm. **h** Representative images of double-immunostaining for CD31 (red) and NG2 (green) in tumor sections from Scr (left), ALDH1A1KD (center) or ALDH1A1^+^ (right) mice. DAPI staining is blue. Magnification 40x. Scale bars, 50 μm

The revised Fig. 3 is given hereafter which includes 3d, 3e, 3f, 3g, 3h, 3i and 3j:

The revised Fig. 4 is given hereafter which includes 4f and 4 g:

The revised Fig. 6 is given hereafter which includes 6d, 6e, 6f, 6g and 6h:
