# Supplementary material for: Stemness marker ALDH1A1 promotes tumor angiogenesis via retinoic acid/HIF-1α/VEGF signalling in MCF-7 breast cancer cells
Source: J Exp Clin Cancer Res. 2018 Dec 12;37:311. doi: 10.1186/s13046-018-0975-0 (PMC6291966; doi:10.1186/s13046-018-0975-0)
Supplement: Supplementary file 1 — Table S1. List of qPCR primers. (PDF 142 kb) [file 13046_2018_975_MOESM1_ESM.pdf]

Table S1. List of qPCR primers

NAME	Forward primer (5’-3’)	Reverse primer (5’-3’)
VEGF	CAAGACAAGAAAATCCCTGTGG	CCTCGGCTTGTCACATCTG
HIF-1α	CCAGTTACGTTCCTTCGATCAGT	TTTGAGTTGCGCTTTCA
CAIX	CTTTGAATGGGCGAGTGATT	CAGGAATTCAGCTGGACTGG
SOX2	TTGCTGCCTCTTTAAGACTAGGA	TAAGCCTGGGGCTCAAACT
NANOG	ACATGCAACCTGAAGACGTGTG	CATGGAAACCAGAACACGTGG
OCT-4	ACATCAAAGCTCTGCAGAAAGAACT	CTGAATACCTTCCCAAATAGAACCC
TWIST	AGCTACGCCTTCTCGGTCT	CCTTCTCTGGAAACAATGACATC
RPL19	GATGCCGGAAAAACACCTTG	TGGCTGTACCCTTCCGCTT
