# Supplementary figures and images for: Stemness marker ALDH1A1 promotes tumor angiogenesis via retinoic acid/HIF-1α/VEGF signalling in MCF-7 breast cancer cells
Source: J Exp Clin Cancer Res. 2018 Dec 12;37:311. doi: 10.1186/s13046-018-0975-0 (PMC6291966; doi:10.1186/s13046-018-0975-0)

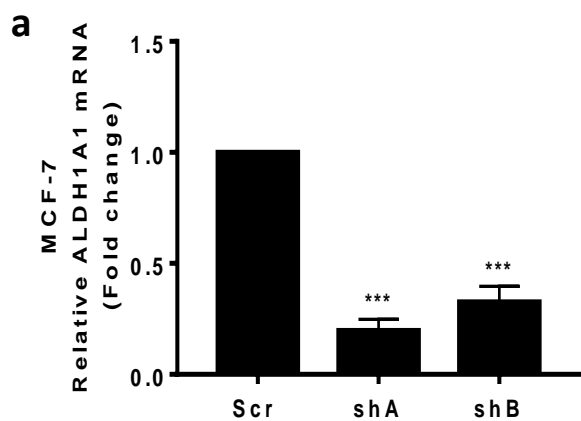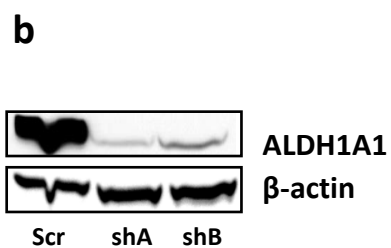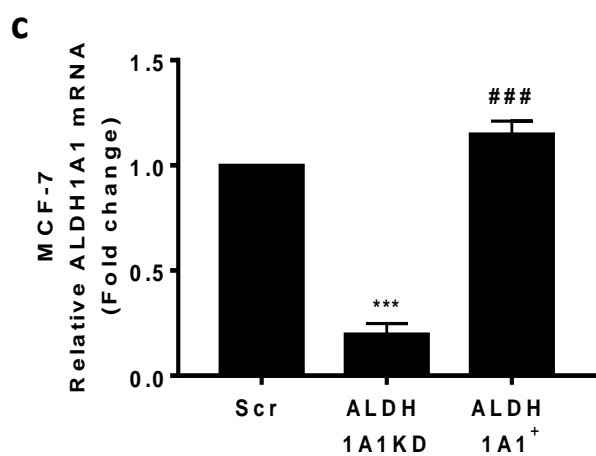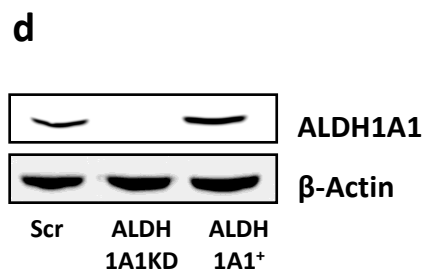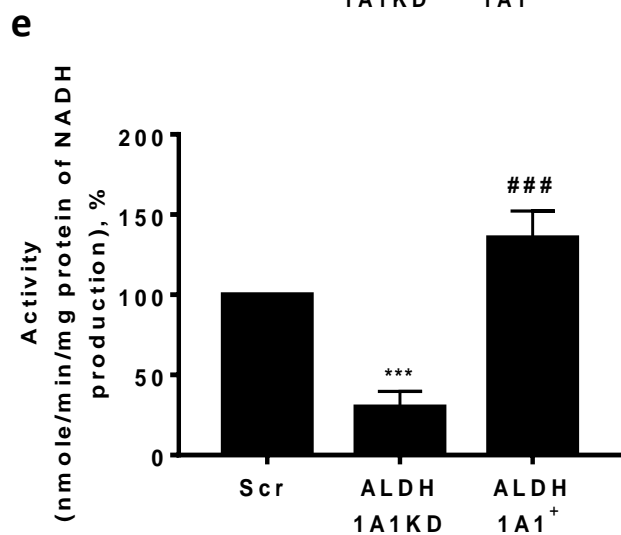

Supplement: Supplementary file 2 — Figure S1. Loss-of function and gain-of function validation to study ALDH1A1 in MCF-7 cells. a. RT-PCR in breast tumor cells (Src and ALDH1A1KD, clones shA and shB) cultured in 10 % FBS for 48 h. ***p < 0.001 vs Scr cells. b. Western blot analysis of breast tumor cells (Src and ALDH1A1KD, clones shA and shB) cultured in 10 % FBS for 48 h. c. RT-PCR analysis of MCF-7 (Src, ALDH1A1KD and ALDH1A1+) cultured in 10 % FBS for 48 h. ***p < 0.001 vs MCF-7 Scr. ###p < 0.001 vs MCF-7 ALDH1A1KD (n = 3). d. Western blot analysis of MCF-7 (Src, ALDH1A1KD and ALDH1A1+) cultured in 10 % FBS for 48 h. β-actin was used as loading control. Gel shown is representative of three experiments with similar results. e. Enzymatic activity in MCF-7 ALDH1A1KD and ALDH1A1+ evaluated by NADH production. Data are reports as in Figure 1. ***p < 0.001 vs MCF-7 Scr. ###p < 0.001 vs MCF-7 ALDH1A1KD. (PDF 372 kb) [file 13046_2018_975_MOESM2_ESM.pdf]

**a**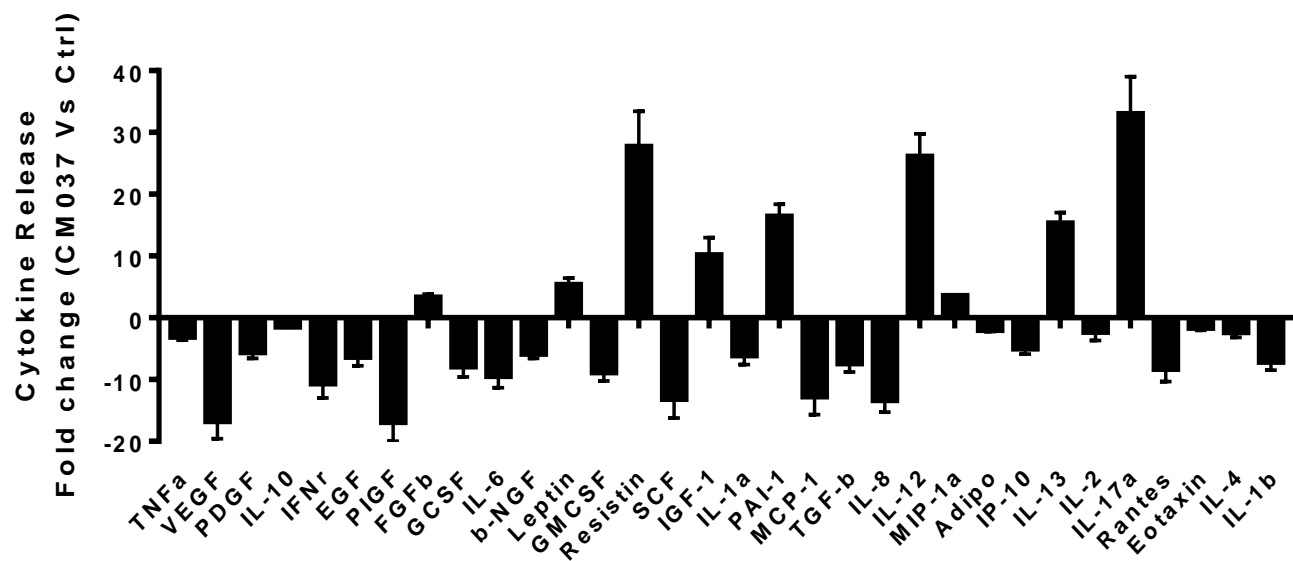**b**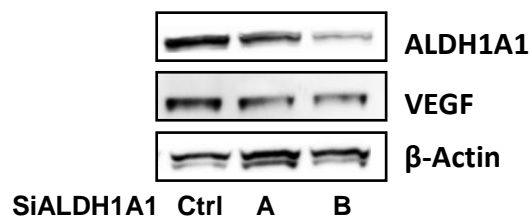

Supplement: Supplementary file 3 — Figure S2. ALDH1A1 activity promotes the release of angiogenic factors in MCF-7. a. Cytokine ELISA plate array in supernatants of MCF-7 treated with CM037 (1 μM) for 48 h. b. Western blot analysis for ALDH1A1 and VEGF on MCF-7 transiently silenced for ALDH1A1 (two sequences of SiRNA, A and B). (PDF 363 kb) [file 13046_2018_975_MOESM3_ESM.pdf]

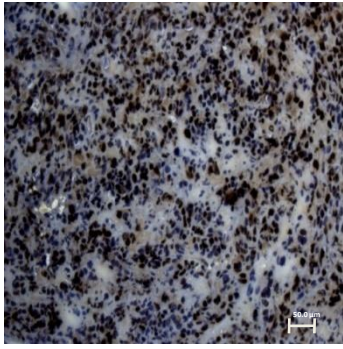

**Scr**

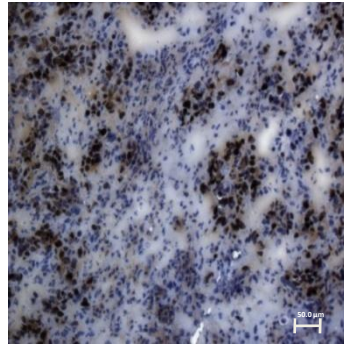

**ALDH1A1KD**

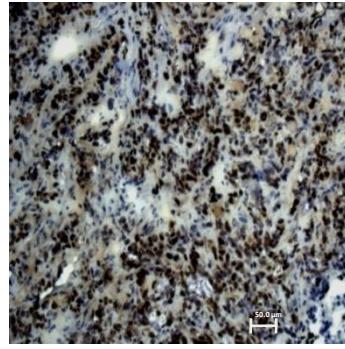

**ALDH1A1<sup>+</sup>**

**Ki67(20x)**

Supplement: Supplementary file 5 — Figure S3. Ki67 index is associated with ALDH1A1 expression in mice tumors. Representative images of immunostaining for Ki67. The number of immunoreactive cells was estimated semi-quantitatively. Tumors ALDH1A1+ and Scr had greater 70 % of positive cells and were scored as +++. Tumors ALDH1A1KD had 10-30 % of positive cells and were scored as +. Magnification 20x. Scale bar, 50 μm. (PDF 298 kb) [file 13046_2018_975_MOESM5_ESM.pdf]
