# Supplementary material for: Stemness marker ALDH1A1 promotes tumor angiogenesis via retinoic acid/HIF-1α/VEGF signalling in MCF-7 breast cancer cells
Source: J Exp Clin Cancer Res. 2018 Dec 12;37:311. doi: 10.1186/s13046-018-0975-0 (PMC6291966; doi:10.1186/s13046-018-0975-0)
Supplement: Supplementary file 4 — Table S2. Angiogenic factors release evaluated by ELISA plate array in supernatants of MCF-7 treated with CM037 (1 μM) for 48 h. (PDF 71 kb) [file 13046_2018_975_MOESM4_ESM.pdf]

Table S2. Angiogenic factors release evaluated by ELISA plate array in supernatants of MCF-7 treated with CM037 (1 µM) for 48 h.

**PROANGIOGENIC** (Fold change CM037 Vs Ctrl)	**ANTIANGIOGENIC** (Fold change CM037 Vs Ctrl)
VEGF (-16.77) ±2.83	IL-12 (+26.12) ± 3.65
PlGF (-16.64) ± 0.96	PAI-1 (+16.41) ± 1.98
IL-8 (-13.39) ± 1.88	
FGFb (+ 3.31) ± 0.52	
